# Modeling seawater intrusion along the Alabama coastline using physical and machine learning models to evaluate the effects of multiscale natural and anthropogenic stresses

**DOI:** 10.1038/s41598-025-06613-6

**Published:** 2025-07-01

**Authors:** Hossein Gholizadeh, T. Prabhakar Clement, Christopher T. Green, Geoffrey R. Tick, Alain M. Plattner, Yong Zhang

**Affiliations:** 1 Department of Geological Sciences, University of Alabama, Tuscaloosa, AL 35487 USA; 2 Department of Civil, Construction, and Environmental Engineering, University of Alabama, Tuscaloosa, AL 35487 USA; 3 Water Resources Mission Area, U.S. Geological Survey, 94035 Moffett Field, CA USA; 4 Groundwater Management Unit, Santa Clara Valley Water District, 95118 San Jose, CA USA

**Keywords:** Seawater intrusion, Physical model, Machine learning, Storm surge, Pumping, Environmental sciences, Hydrology, Geology, Hydrogeology

## Abstract

Seawater intrusion threatens groundwater resources in coastal regions, including southern Baldwin County, Alabama, where the freshwater-saltwater interface dynamics remain poorly understood. To address this gap, this study uses combined physics-based and machine-learning models to quantify seawater intrusion caused by natural (storm surges) and anthropogenic (human activities) perturbations. The long short-term memory network and wavelet analysis were used to assess vertical aquifer vulnerabilities, revealing that the shallow part of the Coastal lowlands aquifer system (CL1) in the southern Baldwin County region is more susceptible to sea level rise and groundwater extraction than deeper aquifers. Based on these findings, a cross-sectional numerical model (physics approach) for the CL1 aquifer was developed to evaluate tidal and storm surge effects, using Tropical Storm Claudette (June 2021) as a case study. Results showed that tidal fluctuations had a minimal impact on the saltwater-freshwater interface location, whereas storm surges caused substantial inland movement, with effects lasting for nine months. The steady-state version of the three-dimensional (3D) physical model predicted seawater intrusion across the entire area, and convolutional neural network-based modeling further validated the model results. The 3D physical model was also applied to a smaller area to assess human impact on the saltwater interface due to two groundwater pumping scenarios (± 50% of the baseline pumping rate). Results revealed that a 50% increase in groundwater withdrawals caused seawater to advance ~ 320 m inland, whereas a 50% reduction led to a ~ 270-meter retreat. This study highlights the vulnerability of Alabama’s shallow coastal aquifers to seawater intrusion due to storm surges and human activities, and demonstrates that combining physics-based models with machine learning approaches can improve groundwater predictions, though its accuracy depends on the availability of site-specific data.

**Supplementary Information:**

The online version contains supplementary material available at 10.1038/s41598-025-06613-6.

## Introduction

Seawater intrusion can threaten the sustainability of groundwater resources, which maintain diverse ecosystems and meet human water demand in coastal regions^1–6^. A recent study by Adams et al.^7^ projected that by 2100, approximately 77% of global coastal areas below 60° north will be affected by seawater intrusion. In Baldwin County, Alabama, seawater intrusion in the coastal region is a pressing issue due to population growth, urban development, local industries, and tourism^8–11^. Dynamics of seawater intrusion and coastal aquifers are further complicated by external forces due to rising sea levels, frequent storm surges, and intensifying hurricanes, whose time scale varies from hours to decades^12,13^. For instance, intensified coastal storms may exacerbate seawater intrusion through storm surge inundation and infiltration^14^. In addition to tidal impact, the local topography substantially influences seawater intrusion dynamics^15^. Accurately quantifying seawater intrusion and groundwater dynamics affected by external forces at various time scales can support the management of coastal aquifers, given their socioeconomic and environmental importance^16–19^.

This study combines a process-based physical model and machine learning approaches to quantify seawater intrusion and groundwater response in a shallow coastal aquifer under external forces across various time scales. The HydroGeoSphere (HGS) model^20^ is selected as the physical model due to its ability to simulate fully coupled surface-subsurface flow and pollutant transport with variable saturation and density, addressing limitations in previous groundwater modeling studies (e.g., Gholizadeh et al.^21^ and Ponprasit et al.^22^). HGS has been successful in modeling seawater intrusion in coastal areas^14,15,23–26^. It is also effective in simulating flow through the vadose zone and capturing storm-induced seawater intrusion^27^.

Long short-term memory (LSTM) proposed by Hochreiter and Schmidhuber^28^ is widely used for long-term time series prediction and has gained prominence in deep learning, as reviewed by Yu et al.^29^. LSTM has been recently applied to quantify groundwater flow and contamination^30–33^. LSTM is therefore selected here as the machine learning approach to model the long-term evolution of groundwater dynamics and its links to various pumping scenarios. In addition, Convolutional Neural Networks (CNN) introduced by LeCun et al.^34^ has proven effective for spatial prediction, offering substantial improvements in applications like flood susceptibility mapping^35,36^.

Notably, traditional numerical models are widely used for seawater intrusion modeling^9,10,18^ but often struggle with computational efficiency and scalability for large-scale, long-term simulations^2,4^. In contrast, deep learning models, particularly LSTM networks and CNNs, effectively capture complex temporal and spatial groundwater patterns^30,31,36^. However, most previous studies^14,15,23,26,27^, including most of those reviewed above, have relied solely on either physical models or machine learning, limiting their ability to integrate mechanistic groundwater flow processes with data-driven insights. This study advances the field by combining HGS, a fully coupled surface-subsurface flow model, with LSTM and CNN to assess seawater intrusion under storm surges and human activities across multiple spatial and temporal scales. The machine learning models were not used to generate input data for the HGS model; instead, each played a distinct and complementary role in the analysis. By leveraging the strengths of both approaches, this hybrid framework is used to enhance predictive capabilities and provides a novel tool to support sustainable groundwater management in vulnerable coastal regions.

The paper is organized as follows. Section “Study site and methodology” introduces the field site and modeling methodology, detailing both the physical and machine learning components. Section “Results” presents the application results. Salinity concentrations, derived from chemical analyses, serve as an indicator of seawater intrusion modeled by HGS. The LSTM model was used to evaluate aquifer dynamics across different depths and time periods, identifying the aquifer most vulnerable to pumping and climatic stress, and the CNN model was employed to validate the HGS simulation results. Wavelet analysis was used to assess correlations between precipitation, sea level, and groundwater level changes. Section “Discussion” examines the effects of natural drivers and human activities on seawater intrusion. HGS was used to simulate the saltwater-freshwater interface along a cross-section under storm surge and tidal conditions. Various groundwater withdrawal scenarios were also considered to assess the influence of human activities. The LSTM model forecasted depth to groundwater (below the land surface) over the next decade under baseline, + 50%, and − 50% pumping scenarios. The HGS model, validated using CNN outputs, was then used to assess the response of the freshwater–saltwater interface to these same pumping conditions over a 10-year simulation period. Section “Conclusions” summarizes the main conclusions.

## Study site and methodology

**Fig. 1:**
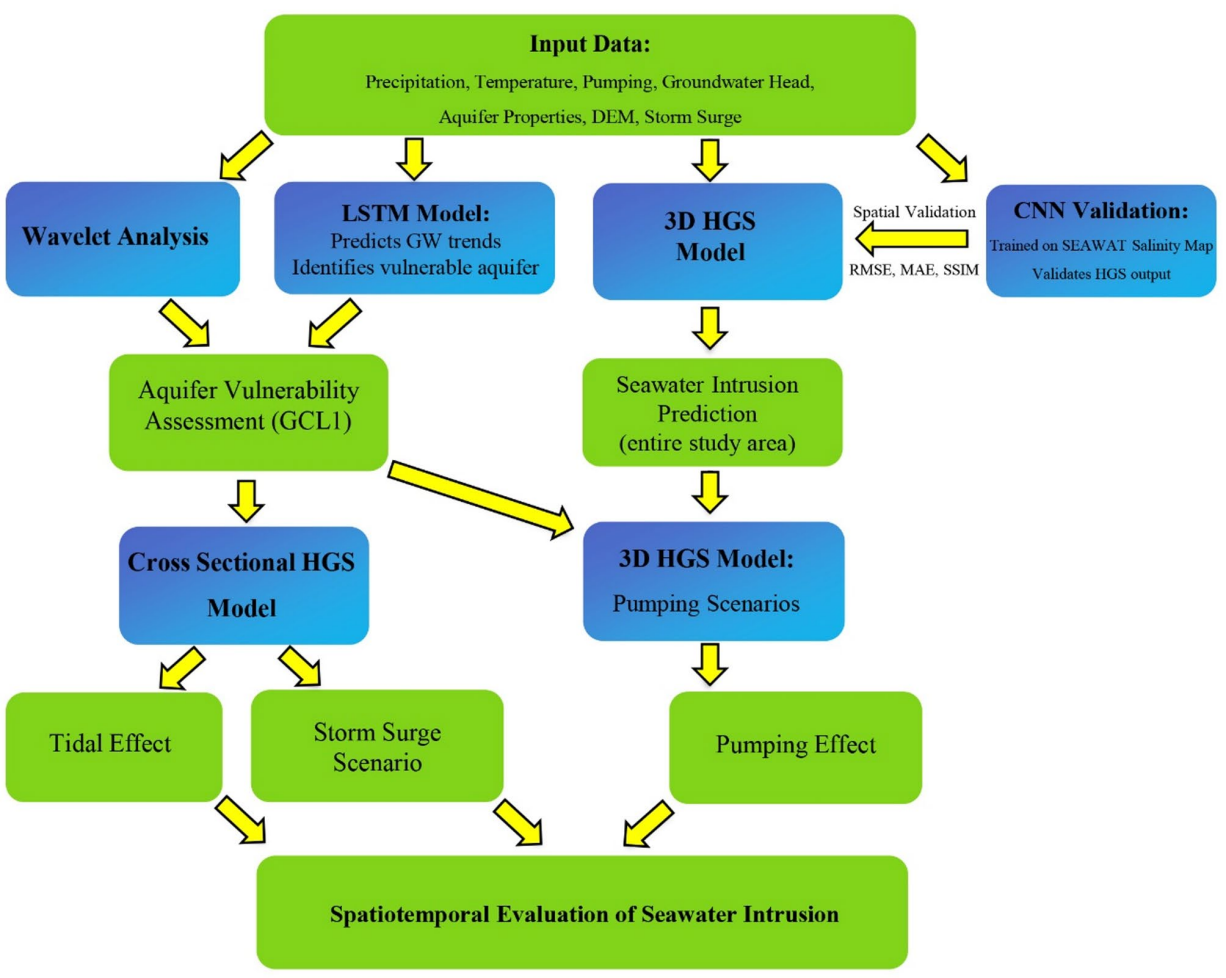
Flowchart summarizing the integrated modeling and analysis framework used to evaluate seawater intrusion in Baldwin County, Alabama. The process begins with the collection of hydroclimatic, geochemical, and geospatial data (top row). These inputs inform four complementary modeling components (subsequent rows): (1) Wavelet analysis to quantify correlations between precipitation, sea level, and groundwater depth over time; (2) a Long Short Term Memory (LSTM) model to assess long-term groundwater dynamics and identify the most vulnerable aquifer; (3) HydroGeoSphere (HGS) simulations to evaluate seawater intrusion under tidal, storm surge, and pumping scenarios; and (4) a Convolutional Neural Network (CNN) model trained on SEAWAT outputs to spatially validate HGS predictions.

This section outlines the study area and the methods used to simulate and analyze seawater intrusion. Figure 1 visually summarizes the methodology and modeling approach used in this study, as briefly described in its caption.

### Study site

**Fig. 2:**
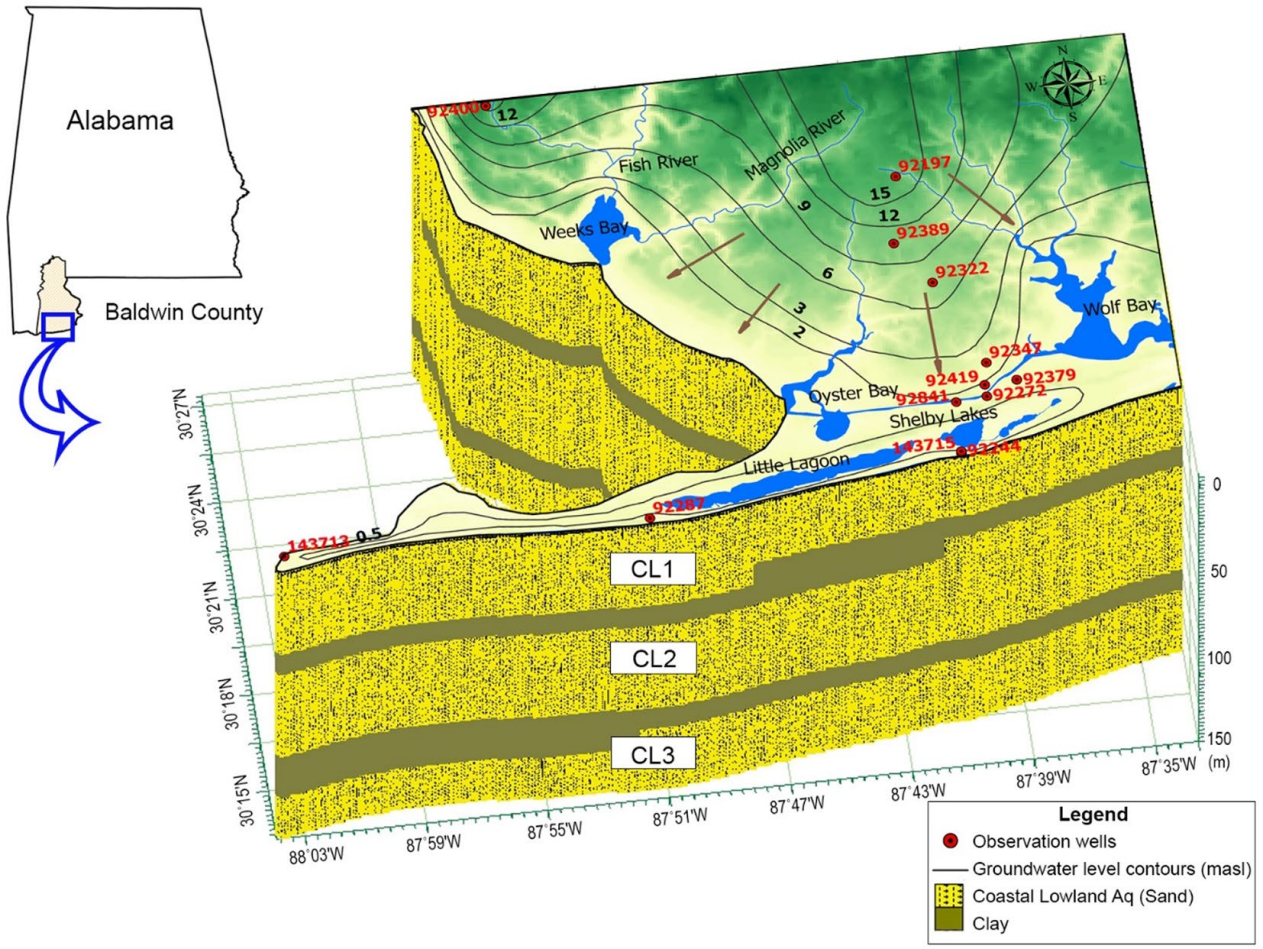
Study area with a cross-sectional fence diagram showing the hydrologic units (the base map is adapted from the USGS). The legend includes red numbers for well locations (15 total; refer to **Table A1**), black numbers for contour lines indicating observed groundwater level (masl), and arrows showing groundwater flow direction. The three vertical aquifers - CL1, CL2, and CL3 - represent hydrogeologic units within the Coastal lowlands aquifer system. The map was created using ArcGIS Pro (v3.5, Esri, https://www.esri.com/en-us/arcgis/products/arcgis-pro/overview) and GMS (v10.2, Aquaveo, https://www.aquaveo.com/software/gms-groundwater-modeling-system-introduction).

This study extends over a 1,274 km^2^ coastal area in southern Baldwin County, Alabama (Fig. 2). This region receives an annual rainfall of 1,724 mm, according to the National Oceanic and Atmospheric Administration (NOAA, https://www.ncei.noaa.gov/cdo-web/datasets, accessed 22 Jan 2025).

The study area has two important rivers: the Magnolia River, with an average streamflow of 1.13 m^3^/s, and the Fish River, at 4.64 m^3^/s (USGS, 2025^37^). Additionally, the site features several notable water bodies, including Weeks Bay, Wolf Bay, Shelby Bay, Oyster Bay, and Little Lagoon, which add to the region’s hydrologic complexity. The area is experiencing rapid population growth, as well as expanding industrial and agricultural activities. According to projections by the Alabama Department of Economic and Community Affairs (ADECA, https://adeca.alabama.gov/water-management/publications-reports-and-information/), water demand between 2010 and 2040 is expected to rise substantially in some areas; for example, an overall increase of 2.5 million gallons per day is expected in the Perdido River subbasin located on the eastern side of the study area. Groundwater serves as an important resource for irrigation, industry, and public supply according to the USGS Water Use Report (https://www.usgs.gov/mission-areas/water-resources/science/water-use-united-states). The regional groundwater flow is depicted in Fig. 2, with black contour lines representing the average groundwater table elevation for unconfined aquifers, derived from 2023 groundwater heads in monitoring wells measured by the Geological Survey of Alabama (GSA, https://www.gsa.state.al.us/gsa/groundwater/realtime), as listed in **Table A1** of the **Supplementary Material** (**SM**). Arrows indicate the direction of horizontal flow. The area contains the sand-based Coastal lowland (CL) aquifer system, consisting of clastic sediments^38^. The regional aquifer system is approximately 150 m thick and has three layers (CL1, CL2, and CL3, from top to bottom) separated by two clay layers (refer to Fig. 2).

### Development of physical, machine learning, and statistical models

#### Long short-term memory model

We used the LSTM model adopted from Neural Hydrology^39^ to build an integrated, data-driven regression framework, using long-term time series of groundwater depth. The LSTM model was trained to forecast groundwater depth and responses to surface inputs in the study area. Our LSTM architecture comprised three layers, with the first two layers containing 20 hidden states each and the third layer forming a dense connection. This dense layer linked the LSTM output from the final time step to a single output neuron using a linear activation function. An additional hyperparameter defined the input sequence length, specifying the number of days of meteorological data required for the LSTM to predict the next groundwater depth. To assess the model’s performance, we employed the Nash–Sutcliffe efficiency (NSE) metric^40^, defined as the complement of the ratio between the error variance of the modeled time series and the variance of the observed time series:

$$\:NSE=1-\frac{\sum_{t=1}^{T}{({Q}_{0}^{t}-{Q}_{m}^{t})}^{2}}{\sum_{t=1}^{T}{({Q}_{0}^{t}-{\stackrel{-}{Q}}_{0})}^{2}},$$

where $\:{\stackrel{-}{Q}}_{0}$ denotes the mean observed groundwater depth, $\:{Q}_{m}^{t}$ and $\:{Q}_{0}^{t}$ represent the modeled and observed groundwater depth at time $\:t$, respectively, and $\:T$ defines the total time. For a perfect model with negligible estimation error variance, the NSE equals 1. Therefore, NSE values closer to 1 indicate stronger alignment between the model’s predictions and actual data. Typically, an NSE exceeding 0.8 is considered a benchmark for acceptable model performance^41^.

We set the sequence length to 365 days, assuming that a full year of seasonality would enable the LSTM to capture the annual cycle. The most recent 365 meteorological measurements (obtained from NOAA (https://www.ncei.noaa.gov/cdo-web/datasets)) were used as inputs for predicting daily groundwater depth. The model was trained for 50 epochs to minimize the root mean square error (RMSE), with an initial learning rate of 1 × 10^−2^. To prevent overfitting, dropout layers were added to the LSTM model^42^ by temporarily deactivating a subset of neurons during training. The optimal epoch count, determined by the highest NSE during the validation period, was found to be 50, achieving the best mean NSE for the training phase. The Adam optimizer^43^ was selected for its efficiency in training time series data. Details of the LSTM model parameters are provided in Table 1.

**Table 1:** LSTM model parameters.

Number	LSTM parameters and values	
1	Number of LSTM layers	2
2	Cell/hidden state length	20
3	Initial forget bias	3
4	Dropout rate	0.4
5	Learning rate	0.01
6	Batch size	256
7	Optimizer	Adam
8	Number of training epochs	50
9	Sequence length	365

The LSTM model was used to identify which aquifer (CL1 or CL2) is more vulnerable to groundwater withdrawal. This analysis guided the selection of the aquifer for subsequent HGS model scenario development. The LSTM model was trained using historical groundwater level data as the target variable. Dynamic inputs included daily precipitation, temperature, and pumping rates, while static inputs consisted of hydrogeological properties such as hydraulic conductivity, soil depth, porosity, and maximum water content (refer to Table 2). The LSTM model is designed to learn temporal relationships directly from observed input–output data. Therefore, processes like aquifer recharge and evapotranspiration are not explicitly calculated; rather, recharge and discharge dynamics are implicitly captured through the correlations between input variables and variations in groundwater levels.

**Table 2:** Required data for LSTM and HGS models.

Model Inputs	Source
Precipitation and temperature	NOAA (https://www.ncei.noaa.gov/cdo-web/datasets)
Groundwater head	GSA (https://www.gsa.state.al.us/gsa/groundwater/realtime)
Hydraulic conductivity, Storage coefficient	Borehole data (https://www.gsa.state.al.us/gsa/groundwater/publications)
Layer thickness	Geological cross sections^38^
Soil depth and porosity and maximum water content	Catchment Attributes and Meteorology for Large-sample Studies (CAMELS) (https://gdex.ucar.edu/dataset/camels.html)
Geochemical data	Water Quality Portal. Washington (DC) : National Water Quality Monitoring Council, United States Geological Survey (USGS), Environmental Protection Agency (EPA); 2021. 10.5066/P9QRKUVJ.
Pumping rate	ADECA (https://adeca.alabama.gov/water-management/publications-reports-and-information/)

#### Wavelet analysis

Wavelet analysis produces a two-dimensional (2D) plot showing variation strengths as a function of period (or frequency) and time. It identifies patterns within specific portions of a time series by comparing them to finite-duration waves, or wavelets^44^. This method was applied to examine relationships between variations in precipitation, sea level, and groundwater depth, addressing a knowledge gap not covered by the LSTM model.

#### HydroGeoSphere model

HGS^45^ was used for numerical modeling of groundwater flow in Baldwin County, Alabama. Main input data for the HGS model included precipitation, pumping rates, and hydrogeological parameters such as groundwater head, hydraulic conductivity, aquifer/aquitard thickness, specific yield, and porosity. Data sources are summarized in Table 2. Mesh grids for the study area were imported into HGS. The grids’ vertical features were defined through layering and the inclusion of surface-water bodies such as Weeks Bay, Wolf Bay, Shelby Bay, Oyster Bay, Little Lagoon, and the Fish and Magnolia Rivers. The model considered three major aquifers (CL1, CL2, and CL3) based on well records^46^, with layer elevations determined from ArcGIS (Esri, Redlands, California) raster files. Based on pumping tests conducted during well completion, as documented in the drillers’ logs^46^, hydraulic conductivity values are as follows: CL1 = 5 × 10⁻⁴ m/s, CL2 = 1 × 10⁻⁴ m/s, and CL3 = 4 × 10⁻⁵ m/s, reflecting a decrease in conductivity with depth. These values were later subject to calibration. A specific yield (S_y_) of 0.3 was assigned to CL1, and storage coefficients for CL2 and CL3 were set at 0.00014 and 0.00006, respectively, based on the pumping test results^46^. Because CL1 may transition between unconfined and confined conditions, an additional storage coefficient of 0.00027 was assigned to it. Surface flow properties, such as soil characteristics and runoff, were derived from remote sensing data and the SWAT model^47–49^. However, HGS was used to integrate core surface domain properties. In this study, surface flow parameters incorporated into the HGS model included a recharge rate of $\:9\times\:{10}^{-4}$ m/day^46^ and Manning’s friction coefficients of 0.1 for drylands and 0.03 for wetlands, adopted from the HGS manual^20^. These parameters were directly imported into the model to support simulations.

Annual average groundwater level data from 11 observation wells (Fig. 2), measured in 2023, were imported into the model to define the water table depth of the unconfined aquifer. The depths of these wells and the aquifers they penetrate are detailed in **Table A1** in **SM**. Boundary conditions for the flow component of the HGS model were established using hydrological data and geological cross-sections from Ponprasit et al.^50^. The southern and western boundaries (Fig. 2) were designed as constant head boundaries (downstream in HGS), while the northern and eastern boundaries were set as variable head boundaries (upstream in HGS). For transport simulation, a salinity concentration of 35,000 mg l^−1^^51^ was assigned at the southern and western boundaries to represent seawater. Groundwater heads measured from 2023 were used to represent steady-state conditions. Additional inputs, including groundwater recharge and discharge, were integrated into the model. These inputs included average rainfall (0.0047 m/day), average pumping rates from active wells (sourced from the latest ADECA records), and recharge data. This comprehensive dataset enabled detailed simulation of groundwater flow dynamics. The model was calibrated using the annual average groundwater head measured in 2023, with the initial seawater intrusion boundary based on previous studies^52,53^. Parameter ESTimation and Uncertainty Analysis (PEST), a model-independent parameter estimation approach introduced by Doherty^54^, was used to perform a sensitivity analysis and identify the parameters most influencing the model output. The PEST results indicated that hydraulic conductivity had the greatest impact on model performance, followed by recharge rate, Manning’s friction coefficient, and specific yield. Considering the study’s focus on seawater intrusion, hydraulic conductivity was prioritized to align simulated and observed groundwater levels while maintaining acceptable accuracy in overland flow depth (difference of less than 10% between observed and simulated values). The calibrated hydraulic conductivity values are 1 × 10⁻³ m/s for CL1, 6 × 10⁻⁵ m/s for CL2, and 1 × 10⁻⁵ ms^−1^ for CL3. Surface flow properties were implemented as variable boundary conditions using time-value tables to represent temporal changes in inputs such as precipitation and surface water flow in the model. A 3D HGS model was developed to simulate the saltwater-freshwater interface across the study area. Longitudinal and transverse dispersivities were set to 100 m and 10 m, respectively, as adopted from^55^. The molecular diffusion coefficient was assigned as 10⁻⁹ m^2^/s based on^20^. These transport parameters were not calibrated with PEST due to the limited availability of salinity time-series data. The transport parameters are summarized in Table 3. In addition, a higher resolution 2D HGS model was constructed to simulate tidal effects and the impact of Tropical Storm Claudette (June 2021) using a cross-sectional approach. The boundary head was defined based on sea level measurements recorded during the storm. A refined 3D HGS model was also developed to assess the influence of groundwater pumping in a focused area. Detailed model setup, including domain configurations, boundary conditions, and implementation assumptions, is presented in Sect. “Cross-sectional HGS model for tidal effects” to “Assessing pumping effects on seawater intrusion using the 3D HGS model”, corresponding to each simulation scenario.

**Table 3:** List of HGS model parameters.

No	HGS parameters and values	
1	Seawater concentration	35,000 mg l^−1^
2	Freshwater concentration	0 mg l^−1^
3	Seawater density	1,025 kg m^−3^
4	Freshwater density	1,000 kg m^−3^
5	Longitudinal dispersivity	100 m
6	Lateral dispersivity	10 m
7	Molecular diffusion coefficient	10⁻⁹ m^2^ s^−1^
8	Porosity	0.3

#### Convolutional neural network model

Given the faster computation speed of machine learning compared to the HGS model (which requires node-by-node calculations and multiple iterations for convergence), we employed the CNN model to generate the saltwater–freshwater interface map across the entire study area for comparison with HGS outputs. In this approach, the CNN was not used as an input to the HGS simulations. Instead, it was implemented as a complementary tool to validate the results of the HGS numerical model. Two input maps were used: a baseline digital elevation model (DEM) was obtained from the USGS (https://www.usgs.gov/national-geospatial-program/national-map) and a seawater intrusion probability map derived from MODFLOW/SEAWAT^56,57^ modeling results. The salinity map generated from SEAWAT simulations, together with the DEM, were used to train the CNN, which was designed to recognize spatial patterns in salinity distribution. Each pixel, originally represented as a 1 × 1 matrix with three color channels, was reshaped to fit the CNN input format. The dataset was then split into training (80%) and testing (20%) subsets. The CNN architecture included an input layer, convolutional layer, flatten layer, dense layer, and output layer, designed to classify pixels based on their color values. The Rectified Linear Unit (ReLU) activation function^58^ was selected for the CNN model because of its effectiveness in accelerating training convergence. Model parameters are presented in Table 4. The CNN was trained on the labeled pixel data and validated with the testing set. Once trained, the model predicted labels for all pixels in the map image, with performance evaluated using RMSE.

**Table 4:** List of CNN model parameters.

Number	CNN parameters and values	
1	Optimizer	Adam
2	Loss function	Sparse categorical cross-entropy
3	Evaluation metric	Accuracy
4	Number of CNN layers	5
5	Activation function	ReLU
6	Number of training epochs	50
7	Learning rate	0.01

## Results

### LSTM modeling for groundwater depth’s evolution and response to pumping

**Fig. 3:**
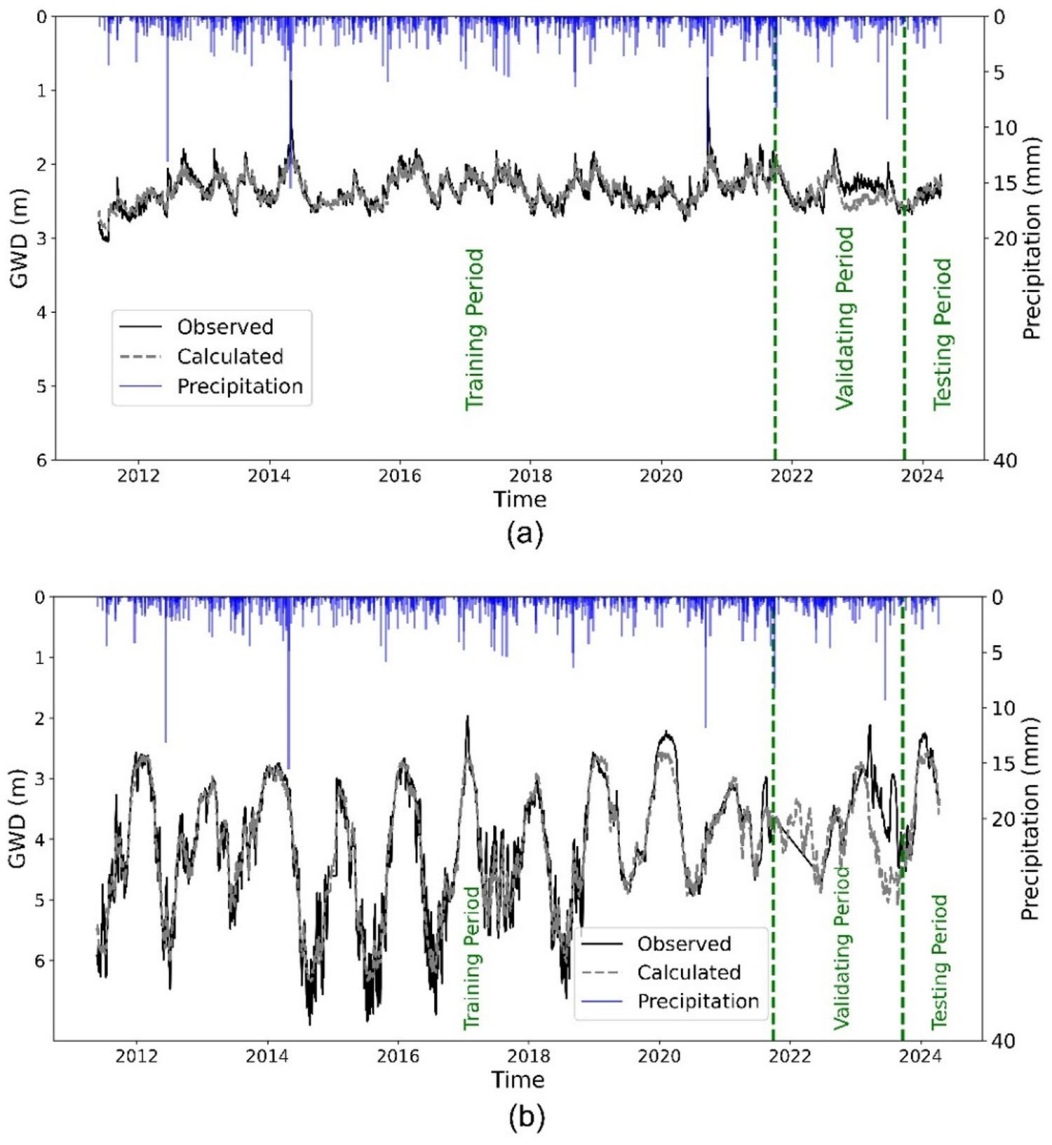
Comparison of LSTM model output with observed groundwater depth for observation wells 92841 (CL1 aquifer) **(a)** and 92272 (CL2 aquifer) **(b)**. The model achieved NSE values of 0.75 and 0.89 during the testing period for CL1 and CL2, respectively. The $\:y$-axis represents groundwater depth (GWD) in meters below land surface.

To predict temporal changes in groundwater depth in CL1 and CL2, the LSTM model described in Sect. “Long short-term memory model” was trained on groundwater depth data from 2011 to 2021, validated on data from 2021 to 2023, and tested on data from 2023 to 2024. A sequence length of 365 days and 50 training epochs were used. The results, shown in Fig. 3, align closely with observed groundwater depths for wells 92841 and 92272. Additionally, scatterplots comparing simulated and observed groundwater depth, along with R² values, are presented in **Fig. A1** in **SM**. The locations of these wells are shown on Fig. 2.

**Fig. 4:**
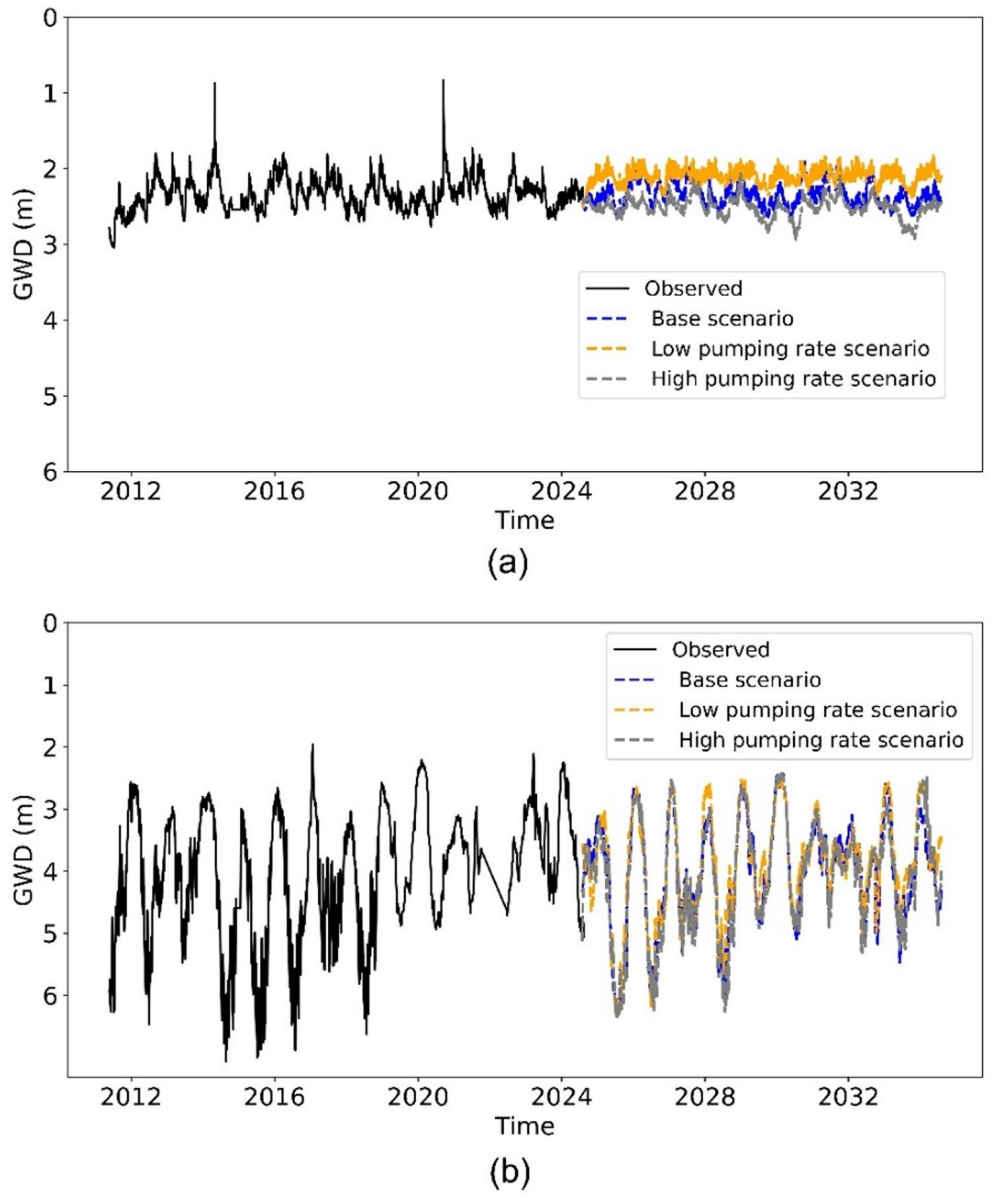
LSTM forecasts of groundwater depth (in meters below land surface) for wells 92841 (CL 1) **(a)** and 92272 (CL 2) **(b)** under different pumping scenarios.

To assess the vulnerability of aquifers at different depths (CL1 and CL2) and the long-term impact of pumping rates on groundwater sustainability, we applied the LSTM model to forecast changes in groundwater depth over the next decade. The LSTM model architecture, as detailed in Sect. “Long short-term memory model”, was unchanged, except for incorporating well pumping rates as a dynamic input. The model was trained on groundwater depth data (depth to the water table from the wellhead) from 2011 to 2024. For the forecast period (2024–2034), we assumed that climate trends from the past decade would persist and simulated three pumping scenarios: the base scenario (no change in the pumping rate), a low pumping rate scenario (50% reduction), and a high pumping rate scenario (50% increase). The forecasted groundwater depth for wells 92841 (screened in aquifer CL1) and 92272 (screened in aquifer CL2), shown in Fig. 4, reveal differing responses to these scenarios. For CL1, the average difference in groundwater depth between the base scenario and the low pumping scenario is 0.70 m, whereas the difference between the base scenario and the high pumping scenario is 0.51 m. For CL2, these differences are 0.37 m and 0.15 m. These results indicate that the shallow aquifer (CL1) is more vulnerable and exhibits greater sensitivity to pumping rate changes than the deeper aquifer (CL2).

### Wavelet analysis of groundwater depth response to precipitation and sea level changes

**Fig. 5:**
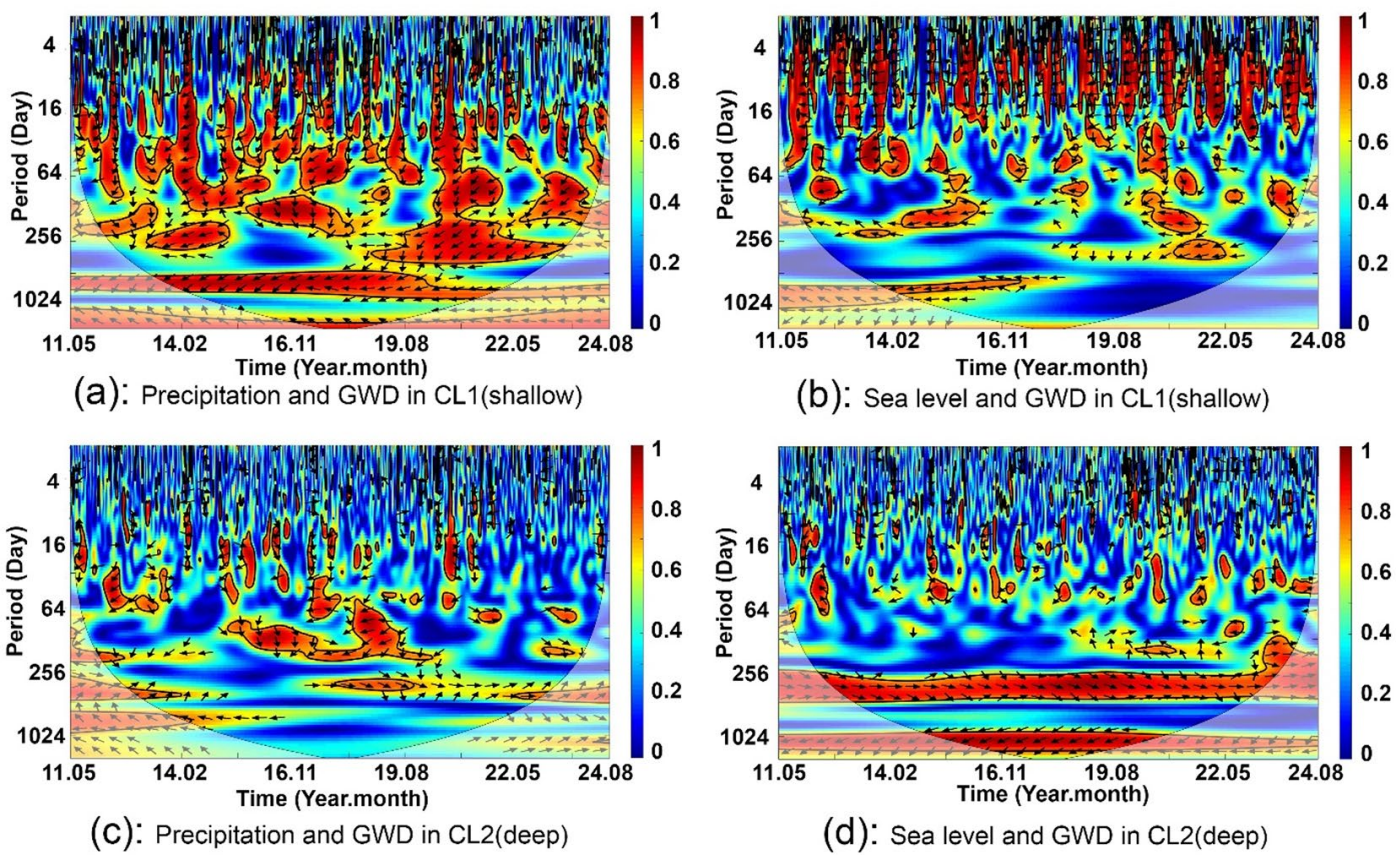
Wavelet coherence power spectrum between precipitation and groundwater depth (GWD) and between sea level and groundwater depth for well 92841 (screened in the shallow aquifer CL1) **(a and b)** and well 92272 (screened in the deep aquifer CL2) **(c and d)**, respectively. Values closer to 1 indicate a stronger correlation (i.e., higher power) between the two signals at a given time and frequency.

To investigate the lag time between sea level changes and groundwater variations in the shallow (CL1) and deep (CL2) aquifers and to determine which is more vulnerable to precipitation recharge, we used wavelet analysis to assess the response of groundwater depth in wells 92841 (CL1) and 92272 (CL2) to precipitation and sea level changes (Fig. 5). For well 92841, the wavelet coherence spectrum between precipitation and groundwater depth exhibits continuous periodic behavior at large temporal scales (512 ~ 1,024 days) and seasonal patterns at smaller scales (16 ~ 128 days) (Fig. 5a). On an annual scale, discontinuous periodic behavior likely reflects variations in annual precipitation, such as cycles of wet and dry years. Additionally, the wavelet coherence power spectrum between sea level and groundwater depth for well 92841 indicates discontinuous periodic characteristics at scales of 4–32 days, corresponding to spring and neap tidal influences (compared to local tidal records), and fragmented periodic behavior at scales of 64–512 days (Fig. 5b).

In contrast, for well 92272, the coherence spectrum between precipitation and groundwater depth shows periodic behavior at monthly and seasonal scales, primarily from 2015 to 2019 (Fig. 5c). The spectrum between sea level and groundwater depth highlights more continuous periodic behavior at scales of 256–512 days and beyond 1,024 days (Fig. 5d).

A comparison of groundwater responses to precipitation and sea level rise between the well in the shallow aquifer (well 92841) and the well in the deep aquifer (well 92272) reveals that the well in the shallow aquifer exhibits a shorter response period to both factors (precipitation and sea level rise).

### HGS model results: Steady-state groundwater flow and transient saltwater-freshwater interface

**Fig. 6:**
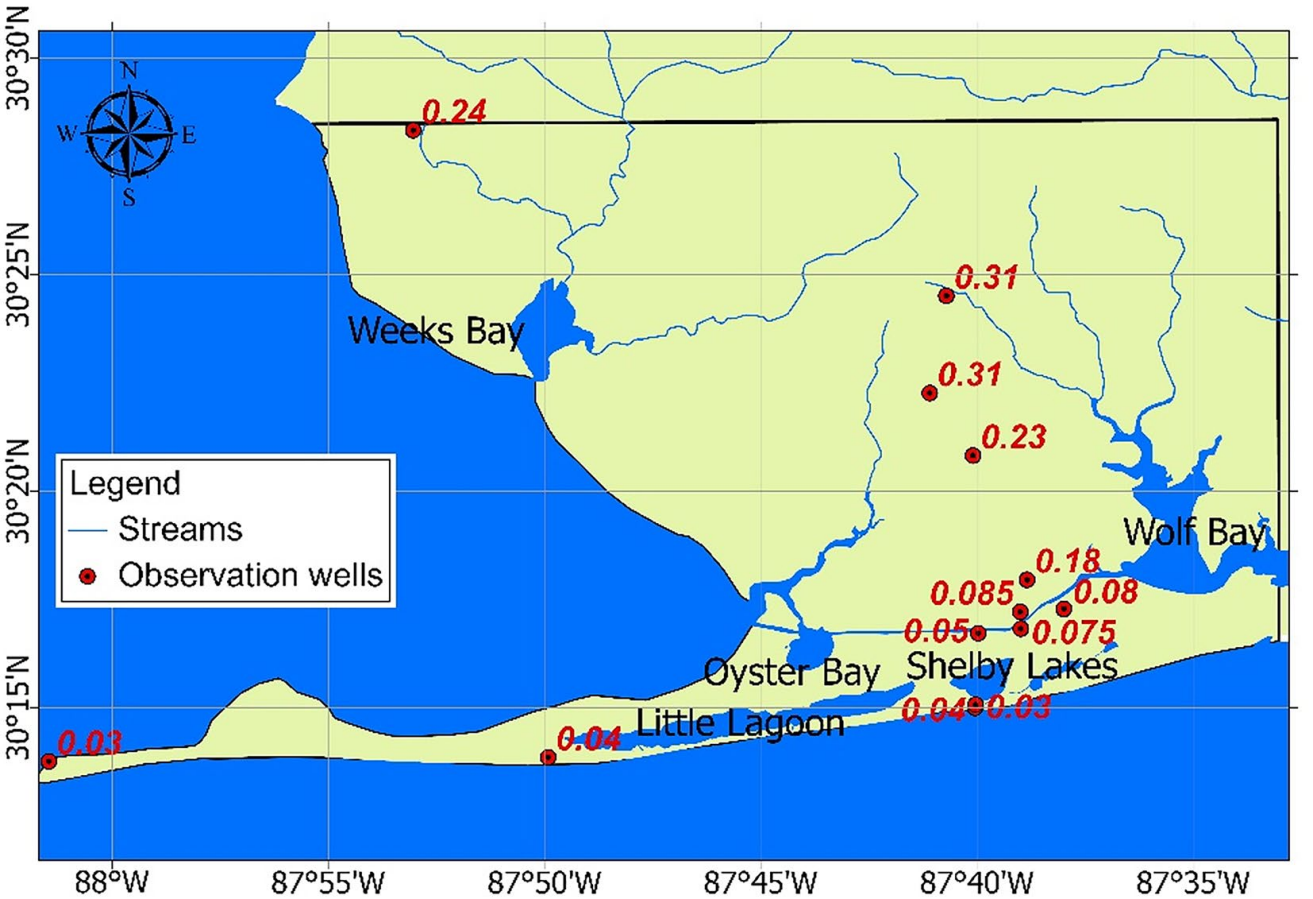
Difference between observed groundwater level (meters above sea level, masl) and those calculated by the HGS model. The map was created using HydroGeoSphere (2023, https://hydrogeosphere.org/) and ArcGIS Pro (v3.5, Esri, https://www.esri.com/en-us/arcgis/products/arcgis-pro/overview).

A 3D HGS model, coupling surface and subsurface steady-state flow, was developed to simulate seawater intrusion and water exchange between surface water and aquifers in the Coastal lowlands aquifer system. The differences between modeled and observed groundwater levels are shown in Fig. 6. According to the groundwater level contour map shown in Fig. 2, groundwater levels in the study area vary up to approximately 15 m. As noted in^59^, the required calibration accuracy depends on the model’s intended use. Given the regional focus on coastal aquifer vulnerability, a difference of less than 0.1 m in these areas - representing less than 10% of the average groundwater head - is considered acceptable. To improve accuracy for flow and transport simulations in coastal areas, we further developed cross-sectional and localized HGS models, as detailed in Sect. “Discussion”. Due to the limited availability and spatial coverage of salinity data, salinity was manually calibrated (focusing on transport parameters, especially the diffusion coefficient) using measured salinity values from the sampling points listed in **Table A2** of the **SM**.

**Fig. 7:**
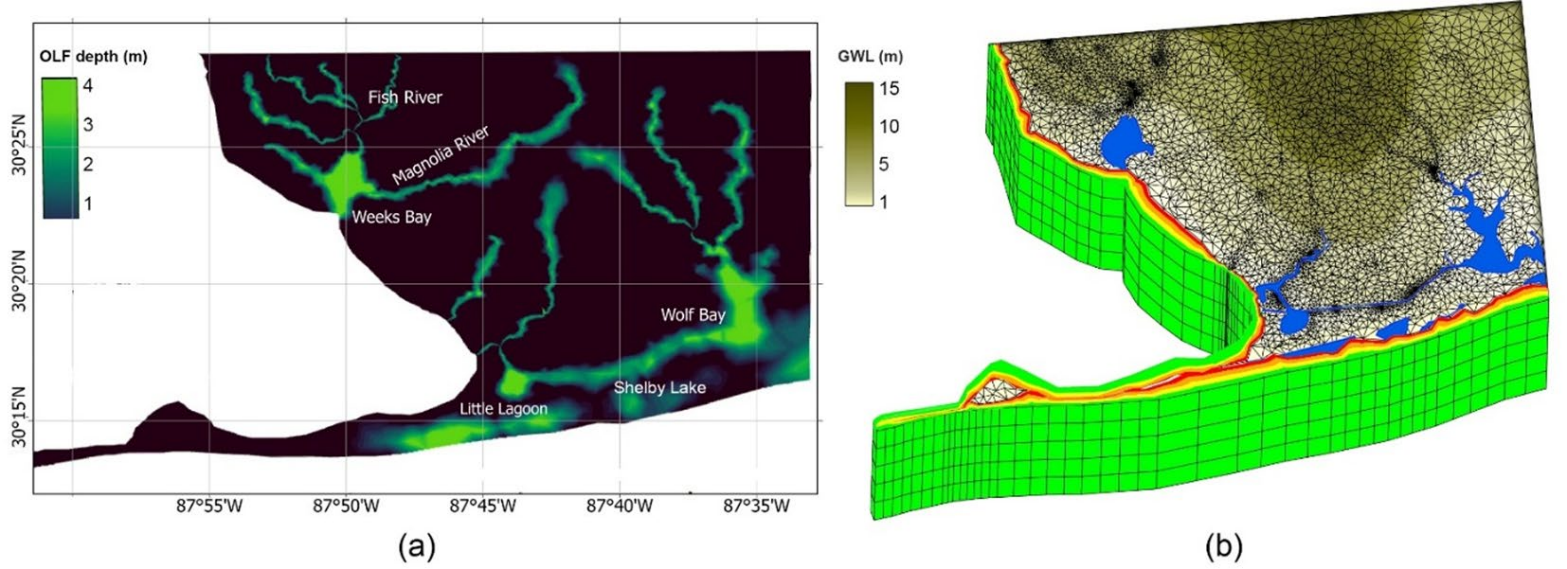
HGS model outputs showing **(a)** overland flow depth and **(b)** snapshots of the saltwater-freshwater interface. The interface is represented by the initial boundary (green), $\:T$= 5 years (yellow), $\:T$ =7.5 years (orange), and $\:T$ =10 years (red, representing the current condition in 2024). The map was created using HydroGeoSphere (2023, https://hydrogeosphere.org/) and Tecplot 360 EX (2021 R1, v2021.1.0.113954, https://tecplot.com/products/tecplot-360/).

Transient simulations were then conducted to assess the dynamics of the saltwater-freshwater interface over time. The results of the 3D seawater intrusion model indicate that the saltwater-freshwater interface has shifted landward since 2013. Figure 7 depicts the temporal variation of seawater intrusion over the past decade, along with the general groundwater flow pattern (Fig. 7b). Notably, areas on the map with chloride concentrations exceed 250 mg/L (approximately 0.05% salinity), the chronic exposure threshold set by the U.S. Environmental Protection Agency^60^, are classified as seawater intrusion zones. The figure also highlights overland flow (OLF) depth across water bodies (Fig. 7a).

### CNN model for the saltwater-freshwater interface

**Fig. 8:**
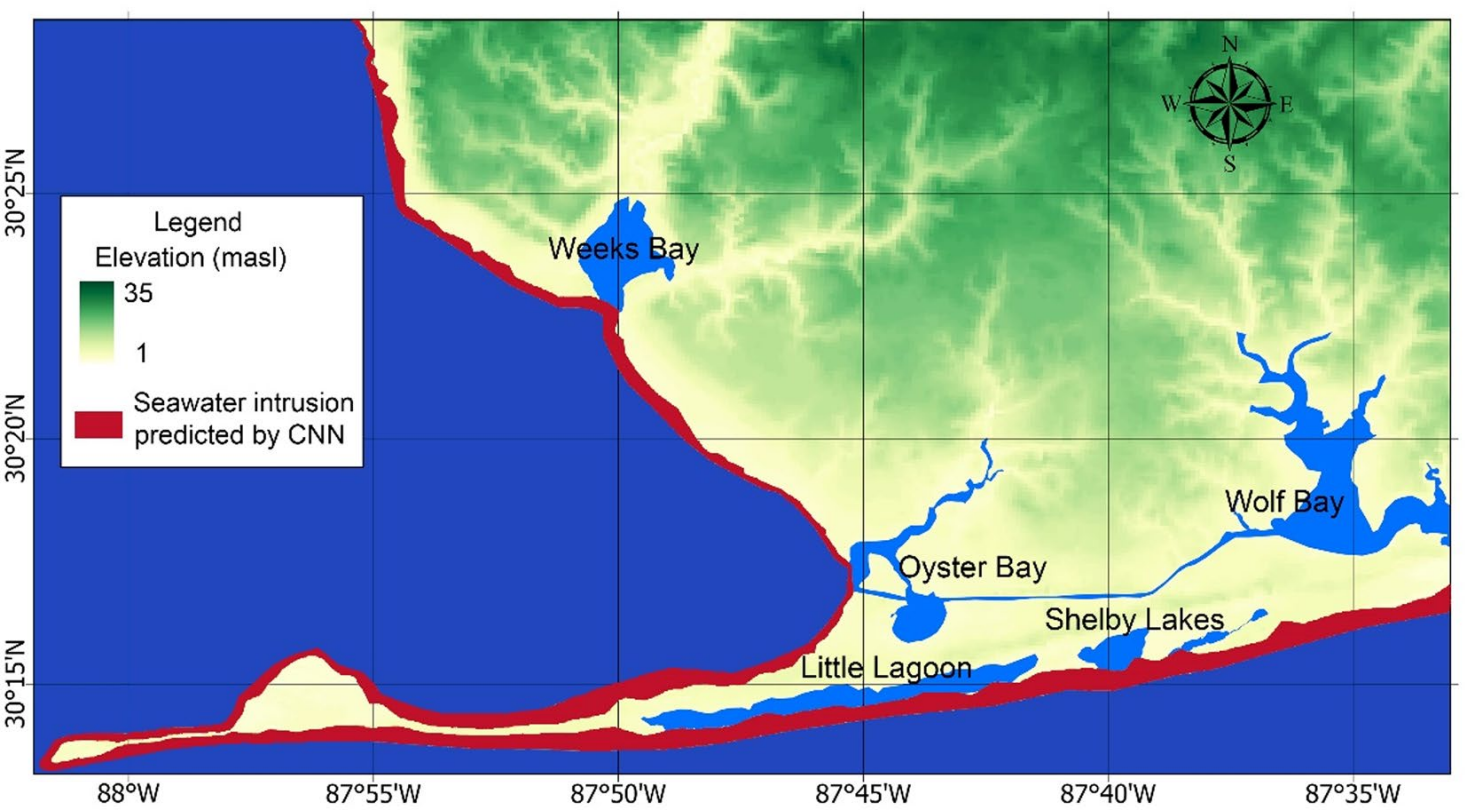
CNN model predictions. The red area represents the predicted extent of seawater intrusion. Elevation values (in meters above sea level, masl) indicate ground surface elevation derived from the DEM (National Elevation Dataset (NED) at 10-meter resolution by the USGS). The map was created using ArcGIS Pro (v3.5, Esri, https://www.esri.com/en-us/arcgis/products/arcgis-pro/overview) and Python 3.11 (https://www.python.org/) with the Matplotlib library (https://matplotlib.org/).

To validate the 3D HGS model presented in Sect. “HGS model results: Steady-state groundwater flow and transient saltwater-freshwater interface”, we developed a CNN model to generate a salinity distribution map as a spatial proxy for seawater intrusion across the entire study area.The CNN model achieved a training accuracy of 0.998 and a loss of 0.0022, where accuracy represents the proportion of correctly classified pixels, and loss measures prediction error using sparse categorical cross-entropy. These metrics were used to evaluate model performance. Figure 8 shows the CNN model’s predictions of the saltwater-freshwater boundary, with an RMSE of 0.157. RMSE, calculated as $\:RMSE=\:\sqrt{\frac{1}{N}\sum\:_{i=1}^{N}{({y}_{i}-{\text{y}}_{\text{p}\text{r}\text{e}\text{d},\text{i}})}^{2}}$, measures the average magnitude of prediction error, where $\:{y}_{i}$ represents the actual value, $\:{y}_{\text{p}\text{r}\text{e}\text{d},\text{i}}$ is the predicted value by the CNN model, and $\:N$ is the total number of pixels. A lower RMSE indicates better model performance by keeping errors on the same scale as the input data.

The CNN model was trained using salinity maps generated from SEAWAT simulations and the DEM. These inputs allowed the model to learn spatial patterns in salinity distribution, including the position of the saltwater-freshwater interface, across the study area. The CNN model’s predictions validate the HGS outcomes, with validation metrics summarized in Table 5. To compare the HGS and CNN models, which output spatial salinity distributions, we used RMSE, mean absolute error (MAE), and the structural similarity index measure (SSIM) to evaluate the predicted salinity maps on a pixel-by-pixel basis. These metrics provide complementary evaluations: RMSE and MAE quantify absolute differences, whereas SSIM evaluates spatial structure and texture similarity, which is important for image-based outputs. The results demonstrate strong agreement between the HGS and CNN predictions. In these metrics, the MAE is calculated as:

$$\:MAE=\frac{1}{N}\sum\nolimits_{i=1}^{n}\left|{y}_{\text{C}\text{N}\text{N},i}-{\text{y}}_{\text{H}\text{G}\text{S},\text{i}}\right|,$$

where $\:N$ is the total number of pixels. MAE quantifies the average absolute difference between the HGS predictions and CNN results, offering a direct and interpretable measure of similarity. Smaller MAE values indicate a closer match between the two models. Additionally, the SSIM is used to assess structural similarity between the HGS simulation results ($\:x$) and the CNN predictions ($\:y$). SSIM is computed as:

$$\:SSIM\left(x,y\right)=\frac{(2{\mu\:}_{x}{\mu\:}_{y}+{C}_{1})(2{\sigma\:}_{xy}+{C}_{2})}{({y}_{x}^{2}+{y}_{y}^{2}+{C}_{1})({\sigma\:}_{x}^{2}+{\sigma\:}_{y}^{2}+{C}_{2})},$$

where $\:{\mu\:}_{x}$ and $\:{\mu\:}_{y}$ are the mean intensities of $\:x$ and $\:y$, respectively, and $\:{\sigma\:}_{x}^{2}$ and $\:{\sigma\:}_{y}^{2}$ are their variances. The term $\:{\sigma\:}_{xy}$ represents the covariance between $\:x$ and $\:y$, and $\:{C}_{1}$ and $\:{C}_{2}$ are small constants added to prevent division by zero. The SSIM value of 0.963, being close to 1, confirms the structural consistency of the HGS predictions with the CNN results, demonstrating a strong agreement between the two models.

**Table 5:** Validation metrics for HGS and CNN Results.

Number	Metric	Value
1	RMSE	0.157
2	MAE	0.025
3	SSIM	0.963

## Discussion

In this study, the LSTM model was used to predict long-term variations in coastal groundwater levels, enabling the identification of the aquifer most vulnerable to groundwater withdrawal. Based on these findings, high-resolution HGS models were developed to assess the effects of tidal fluctuations, storm surges, and pumping on seawater intrusion. Sensitivity analysis using PEST ranked the main flow parameters as follows: hydraulic conductivity, recharge rate, Manning’s friction coefficient, and specific yield. Transport parameters (e.g., dispersion coefficients, molecular diffusion) were adopted from the literature on similar aquifers due to the limited availability of salinity time-series data. This highlights the importance of reliable long-term, observation networks of groundwater and seawater could allow for better estimates of aquifer flow parameters and salinity data which would facilitate more accurate baselines to support modeling. Given the scarcity of observed salinity data, CNN model outputs were used to validate the 3D HGS simulation results across the entire study area. The performance metrics (RMSE and MAE; refer to Table 5) demonstrated strong agreement between HGS and CNN results. Although limited by data constraints, this approach supports a semi-quantitative interpretation of the modeled seawater intrusion patterns. Based on the LSTM and wavelet analysis results presented above, the shallower aquifer CL1 was identified as more vulnerable to seawater intrusion caused by natural events like sea-level rise and anthropogenic activities such as groundwater withdrawals. Therefore, this study focuses on CL1 to further explore the impact of various stressors on seawater intrusion using the HGS model. We concentrated on cross-sectional and localized HGS models to achieve more precise results for flow and transport.

### Cross-sectional HGS model for tidal effects

**Fig. 9:**
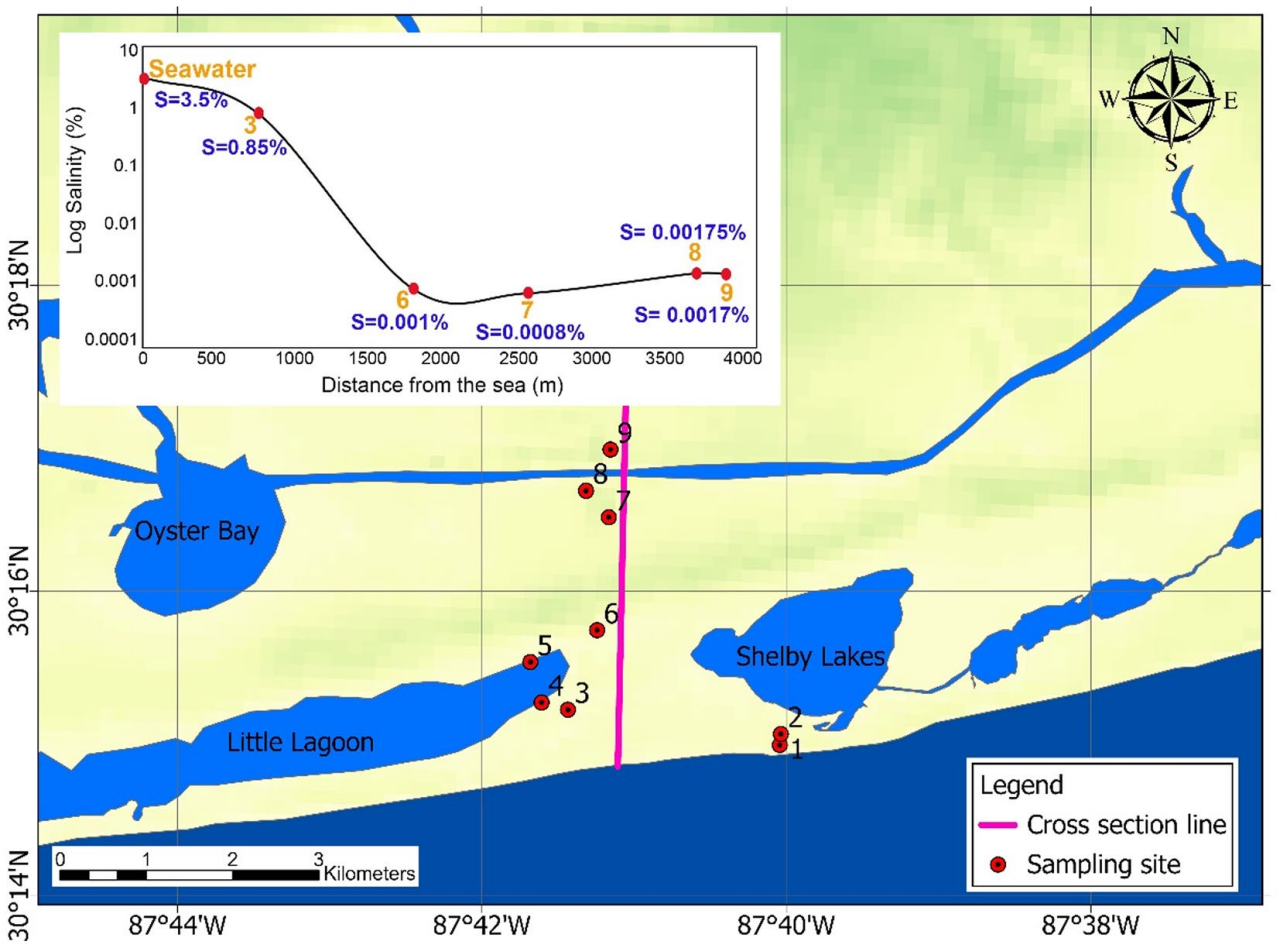
Spatial distribution of wells with salinity data (refer to **Table A2**), showing a decreasing salinity trend with distance from the coast (the base map is adapted from the USGS). The map was created using ArcGIS Pro (version 3.5, Esri, https://www.esri.com/en-us/arcgis/products/arcgis-pro/overview).

A cross-sectional HGS model was developed using salinity data from Huettemann^61^ and our collected data to provide a high-resolution analysis of seawater intrusion near the coastline. Figure 9 shows the cross-section location and observation wells used for groundwater salinity measurements. The salinity-distance graph illustrates a decreasing salinity trend with distance from the coastline, which was used to calibrate the HGS model. Well depths and screen depths for these observation wells are listed in **Table A2** in **SM**. The model domain, measuring 4,000 m in length and 15 m in depth, represents a 2D profile extending inland from the Gulf Coast. It operates under steady-state groundwater flow conditions, with the seaward boundary set as a specified head boundary of either 0.129 m or −0.112 m (the steady-state flow model was run twice), representing the maximum and minimum sea levels above mean lower low water (MLLW) - the average of the lowest low tides recorded over a 19-yr period (https://tidesandcurrents.noaa.gov/datum_options.html). Seawater salinity was set at 3.5% on the coastal side and 0.001% inland. Additional parameters, including recharge rates and aquifer properties, are detailed in Sect. 2.2.3.

**Fig. 10:**
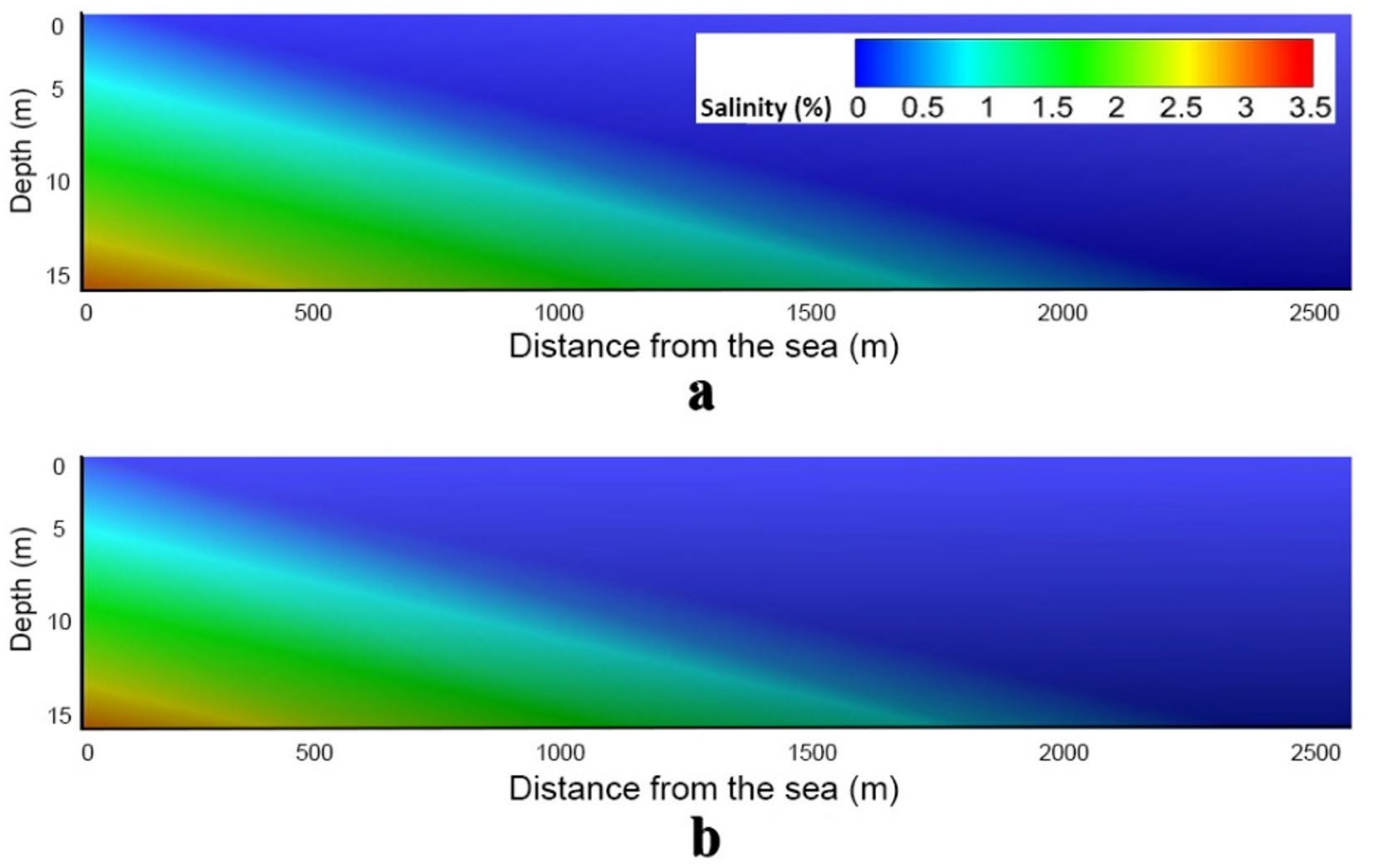
Simulated saltwater-freshwater mixing zone from the HGS model for **(a)** sea level = 0.129 m (maximum tidal head) and **(b)** sea level= −0.112 m (minimum tidal head). The negligible differences between the two figures (too small to illustrate clearly on a difference map) are likely due to the relatively small tidal head range.

Chang et al.^62^ showed that sea-level rise would lift the entire aquifer, mitigating the long-term impacts of seawater intrusion. In contrast, LeRoux et al.^14^ examined tidal effects in mega-tidal systems (tidal ranges exceeding 8 m) and during tidal-surge phases (interactions between tidal and storm surge processes). Their findings showed that sea-level rise and storm surges drive the landward migration of the saline plume, leading to the salinization of freshwater resources. Here, we investigate annual tidal effects using the HGS model, incorporating maximum and minimum sea levels from 2023. **Fig. A2** displays long- and short-term sea level variations based on NOAA’s National Ocean Service data (https://tidesandcurrents.noaa.gov/sltrends/sltrends_station.shtml?id=8735180). The results indicate that tidal effects have a negligible impact on the saltwater-freshwater transition zone (Fig. 10), likely due to the low maximum tidal head (0.129 m) in the study area. Additional model tests (not shown) suggest that tidal ranges exceeding 0.3 m could influence the freshwater-saltwater interface by altering hydraulic gradients, with the final extent of the impact depending on local hydrogeological conditions and aquifer permeability. For example, high-permeability zones are more sensitive to even moderate tidal changes^63^.

### Storm surge impact on seawater intrusion

**Fig. 11:**
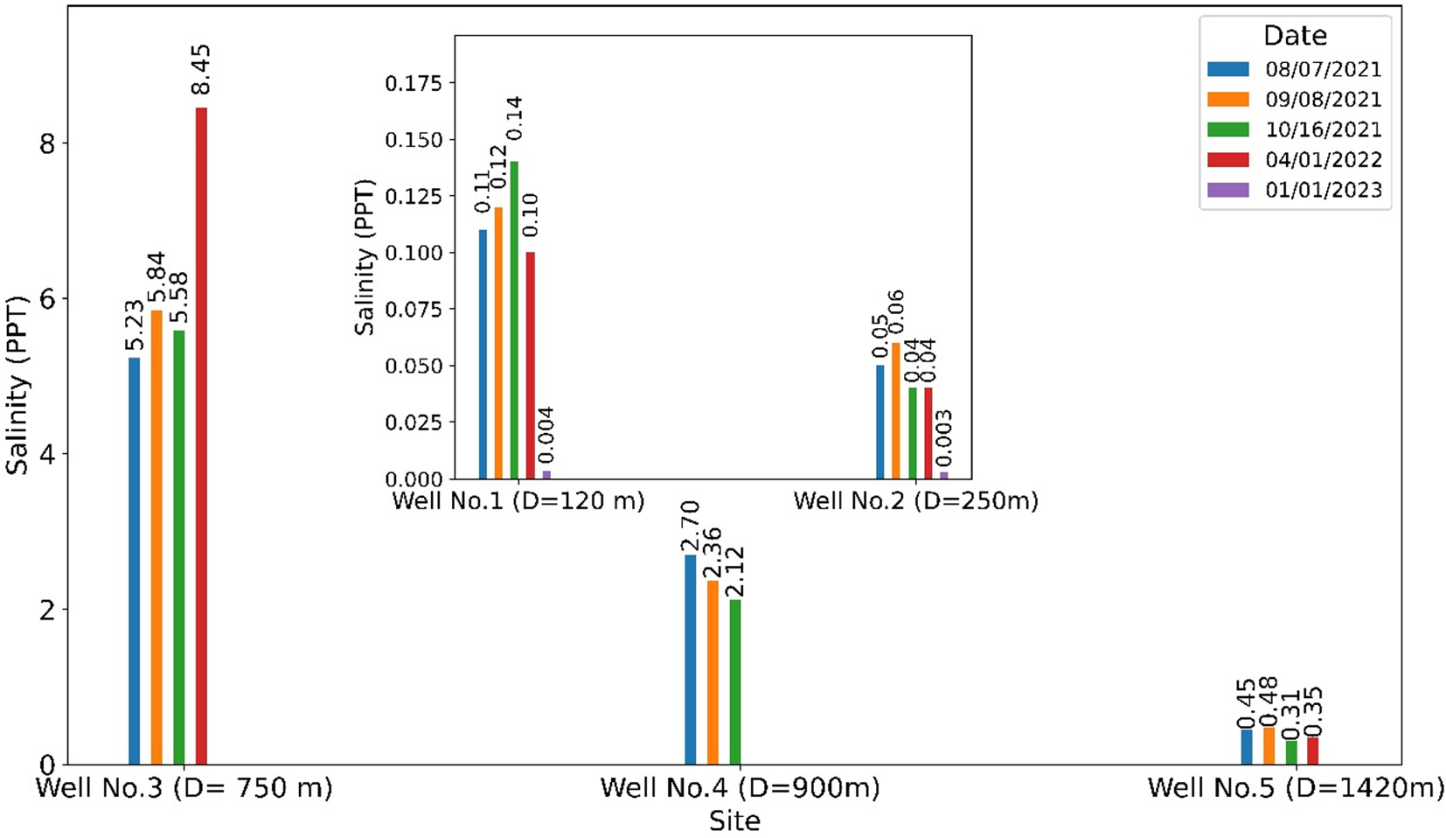
Salinity measurements (PPT) at wells in the study area (refer to **Table A2**). The small purple bar charts on 01/01/2023 for Well No.1 and Well No.2 represent normal conditions without storm influence, with salinity values of 0.004 and 0.003, respectively. “D” is the distance from the sea.

We further used the cross-sectional HGS model, incorporating salinity data from Huettemann^57^ and additional field data (Fig. 9), to assess the storm surge impact on seawater intrusion near the coastline. Tropical Storm Claudette impacted southern and coastal Alabama on June 19, 2021, with substantial effects reported at Dauphin Island and Fort Morgan (located at the western tip of Gulf Shores on Mobile Point). Surf heights, including surge effects, reached 1.8–3.7 m in Mobile Bay (https://www.weather.gov/mob/claudette). To evaluate the storm’s influence on seawater intrusion, we simulated salinity conditions using data from August 2021, October 2021, and April 2022 (Fig. 11). Salinity trends by distance from the sea (denoted as “D”) in Fig. 11 show a decreasing salinity gradient with increasing inland distance. Notably, wells GS-2 and GS-3 (the two closest to the ocean; referring to Well No. 1 and Well No. 2 in Fig. 9) exhibited low salinity levels and are plotted separately due to their relatively small values. This lower salinity is likely due to their shallow depths (**Table A2**), implying a stronger influence from groundwater recharge via precipitation rather than seawater intrusion. These results highlight substantial differences between the storm surge impact period (August 2021 - April 2022) and normal conditions (January 1, 2023).

**Fig. 12:**
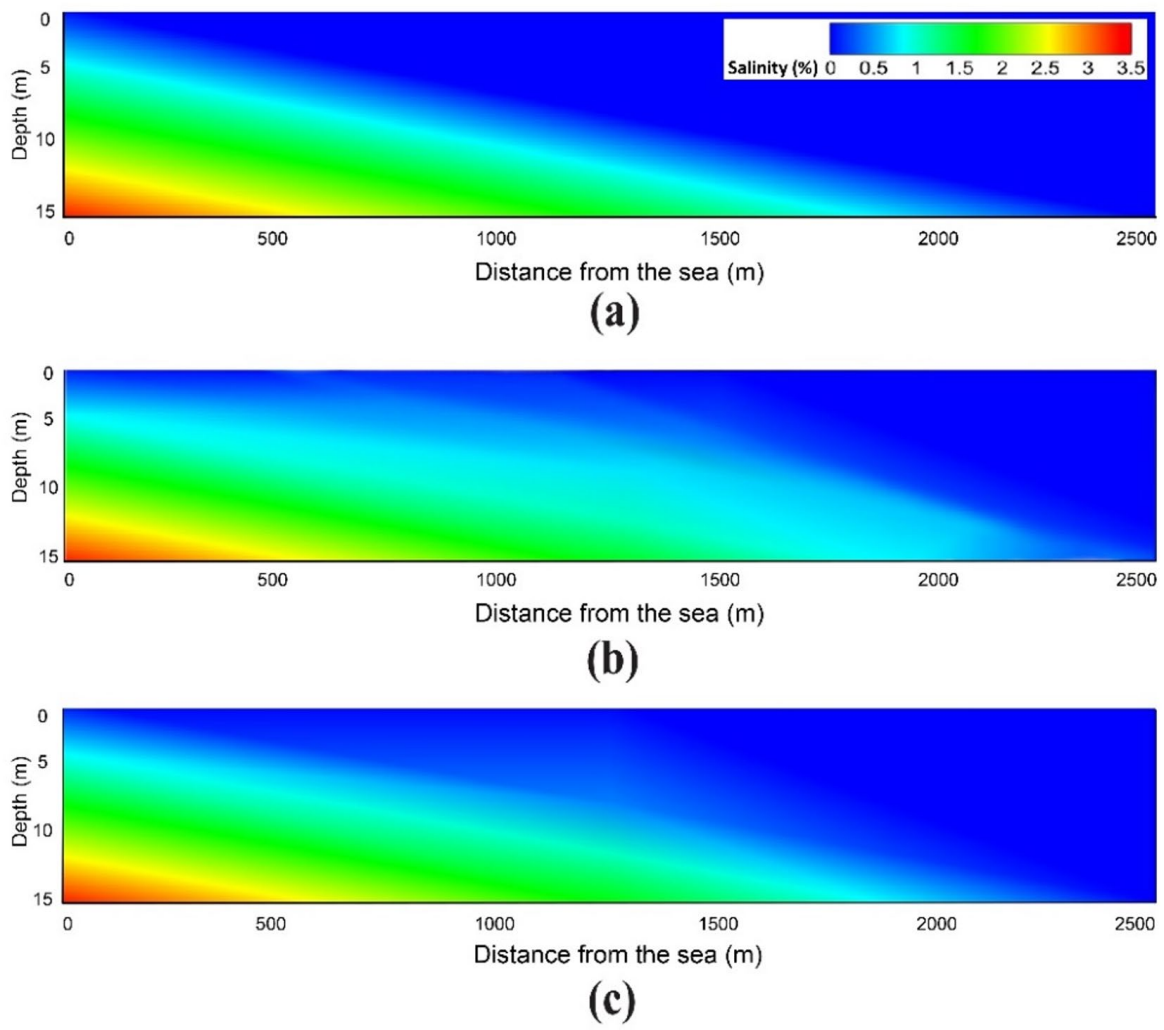
Snapshots of the saltwater-freshwater interface for **(a)** August 2021, **(b)** April 2022, and **(c)** November 2022.

The model domain is identical to that described in Sect. “Cross-sectional HGS model for tidal effects”, with the seaward boundary head set at 0.9 m above MLLW, based on NOAA’s National Ocean Service data for Dauphin Island, Alabama, on June 19, 2021 (https://tidesandcurrents.noaa.gov/waterlevels.html?id=8735180). Figure 12 presents the cross-sectional model results, showing seawater plume movement in April 2022, approximately nine months after Tropical Storm Claudette. The results suggest that storm surges can drive seawater intrusion up to 2,000 m inland along the cross-section under steady-state flow conditions. The observed salinity increase to 0.0012% in April 2022 at the GSPE well ─ located 1,750 m from the coastline (referring to Well No. 6 in Fig. 9), where normal salinity is 0.001% or less ─ is consistent with the model’s results. Therefore, the saltwater-freshwater interface can shift gradually due to long-term sea-level rise and abruptly in response to transient/impulsive events like storm surges. These findings highlight the need to consider both steady-state conditions and transient phenomena when evaluating coastal aquifer systems.

### Assessing pumping effects on seawater intrusion using the 3D HGS model

**Fig. 13:**
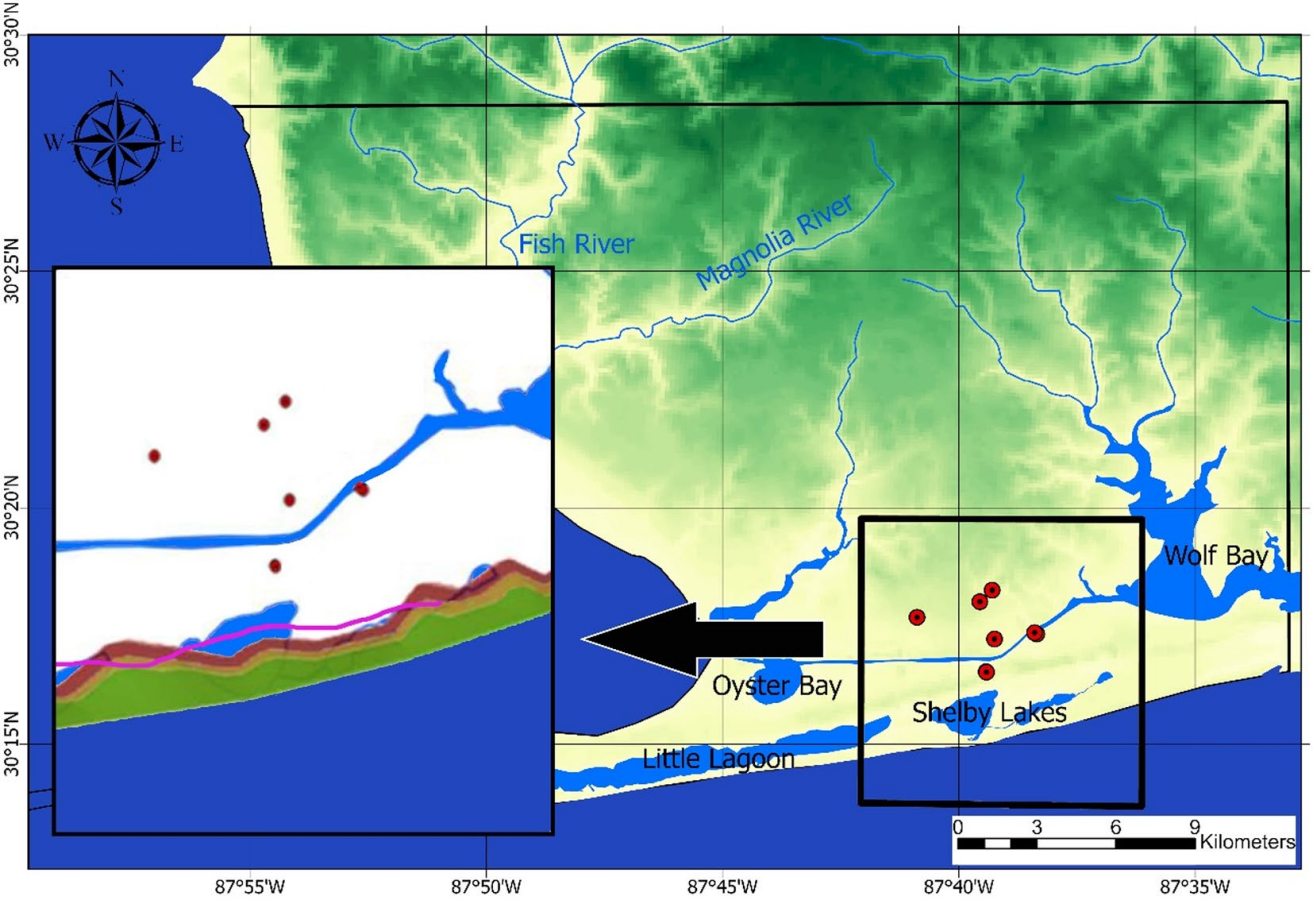
Maximum extent of seawater intrusion (with salinity > 0.05%) at the bottom of the CL1 aquifer for the base scenario (orange), 50% increased pumping rate (red), and 50% decreased pumping rate (green) (the base map is adapted from the USGS). The map was created using HydroGeoSphere (2023, https://hydrogeosphere.org/), Tecplot 360 EX (2021 R1, v2021.1.0.113954, https://tecplot.com/products/tecplot-360/), and ArcGIS Pro (v3.5, Esri, https://www.esri.com/en-us/arcgis/products/arcgis-pro/overview).

Using available pumping well data, we analyzed a localized area to assess how varying pumping rates affect seawater intrusion, measured by the extent of aquifer salinization. Eight pumping wells in the produtive CL1 aquifer were examined, each operating at an average rate of 1,610.5 m^3^/day (sourced from the Alabama Department of Economic and Community Affairs, Office of Water Resources, 2024) in the baseline scenario. To evaluate sensitivity to pumping intensity, we modeled two additional scenarios: a 50% increase and a 50% decrease in pumping rates over 10 years, while keeping precipitation constant. Figure 13 illustrates the HGS modeling results for the three scenarios. Red areas indicate seawater intrusion under increased pumping rates, and green areas represent intrusion under reduced pumping. A 50% increase in pumping advanced the seawater front by 320 m inland over 10 years, whereas a 50% reduction caused a 270-meter retreat toward the sea. The pink line in Fig. 13 marks the 2015 seawater intrusion boundary estimated from Coburn’s (2015) model^64^, which was calibrated using six short (< 200 m) electrical resistivity tomography (ERT) profiles with the MODFLOW/SEAWAT model for Gulf Shores, Alabama. Our HGS results show that seawater intrusion in CL1 extends further into dryland areas than into wetlands with surrounding freshwater bodies, such as Shelby Lakes (marked in Fig. 13).

In summary, our model results indicate that the shallow CL1 aquifer is more vulnerable to seawater intrusion than CL2, as shown by LSTM forecasts and wavelet analysis in Sect. “Results”. Although tidal variations had minimal impact, storm surges substantially pushed the intrusion boundary inland, with effects persisting nine months post-surge. The 3D HGS model, simulating three pumping scenarios, revealed that higher pumping rates could accelerate inland movement of the saltwater front.

## Conclusions

Seawater intrusion, driven by both natural and anthropogenic factors, could affect potable groundwater in Baldwin County, Alabama. Despite its importance, the freshwater-saltwater interface dynamics in this region remain poorly understood since the influence of these factors has not been systematically quantified. To address this knowledge gap, we used both physics-based models and machine learning approaches to assess seawater intrusion under tidal, storm surge, and human influences across various spatiotemporal scales. LSTM networks and wavelet analyses identified the shallow CL1 aquifer as the most vulnerable to precipitation and pumping compared to deeper aquifers. A cross-sectional HGS model for CL1, calibrated using observed salinity levels assembled from literature data and our field sampling efforts, showed negligible annual tidal effects but substantial storm surge impacts, with seawater intrusion persisting up to nine months post-surge event. Additionally, a 3D HGS model under steady-state conditions predicted seawater intrusion across the study area, validated by a CNN model with an acceptable level of accuracy. In a localized area, 3D HGS simulations revealed that a 50% increase in pumping extended seawater intrusion 320 m inland, whereas a 50% reduction led to a 270 m retreat. This study highlights the vulnerability of Alabama’s coastal aquifers to seawater intrusion and demonstrates the potential of combining physics-based numerical and data-based machine learning models for developing approaches to support sustainable groundwater management in coastal regions. Although this model was developed specifically for southern Baldwin County, the framework is transferable to other coastal aquifer systems. Machine learning techniques, such as CNN, can offer computational advantages for predicting seawater intrusion patterns, providing a practical alternative for traditional numerical models. However, the successful application of this framework would depend on the availability of local data, including aquifer characteristics, various water budget components, and long-term water quality records.

## Electronic supplementary material

Below is the link to the electronic supplementary material.

Supplementary Material 1

## Data Availability

Data are available upon request from the corresponding author.

## References

[1] Bear, J., Cheng, A. H. D., Sorek, S., Ouazar, D. & Herrera, I. *Seawater Intrusion in Coastal Aquifers: Concepts, Methods and Practices*Vol. 14 (Springer Science & Business Media, 1999).

[2] Werner, A. D. A review of seawater intrusion and its management in Australia. *Hydrogeol. J.* **1** (18), 281–285. 10.1007/s10040-009-0465-8 (2010).

[3] Pool, M. & Carrera, J. A correction factor to account for mixing in Ghyben-Herzberg and critical pumping rate approximations of seawater intrusion in coastal aquifers. *Water Resour. Res.* **47** (5), W05506. 10.1029/2010WR010256 (2011).

[4] Werner, A. D. et al. Seawater intrusion processes, investigation, and management: recent advances and future challenges. *Adv. Water Resour.* **51**, 3–26. 10.1016/j.advwatres.2012.03.004 (2013).

[5] Costall, A. R. et al. Groundwater throughflow and seawater intrusion in high quality coastal aquifers. *Sci. Rep.* **10** (1), 9866. 10.1038/s41598-020-66516-6 (2020). 32555499 10.1038/s41598-020-66516-6 PMC7300005

[6] Mueller, W. et al. Saltwater intrusion and human health risks for coastal populations under 2050 climate scenarios. *Sci. Rep.* **14** (1), 15881. 10.1038/s41598-024-66956-4 (2024). 38987576 10.1038/s41598-024-66956-4 PMC11237024

[7] Adams, K. H. et al. Climate-induced saltwater intrusion in 2100: Recharge‐driven severity, sea level‐driven prevalence. *Geophys Res. Lett* **51**(22). https://doi.org/10.1029/2024GL110359 (2024). e2024GL110359. 10.1029/2024GL110359 PMC11583115 39582589

[8] Murgulet, D. & Tick, G. The extent of saltwater intrusion in Southern Baldwin county, Alabama. *Environ. Geol.* 55–1245. 10.1007/s00254-007-1068-0 (2008).

[9] Lin, J., Snodsmith, J. B., Zheng, C. & Wu, J. A modeling study of seawater intrusion in Alabama Gulf coast, USA. *Environ. Geol.* **57**, 119–130. 10.1007/s00254-008-1288-y (2009).

[10] Chang, S. W., Nemec, K., Kalin, L. & Clement, T. P. Impacts of climate change and urbanization on groundwater resources in a barrier Island. *J. Environ. Eng.* **142** (12), D4016001. 10.1061/(ASCE)EE.1943-7870.0001123 (2016).

[11] Jasechko, S., Perrone, D., Seybold, H., Fan, Y. & Kirchner, J. W. Groundwater level observations in 250,000 coastal US wells reveal scope of potential seawater intrusion. *Nat. Commun.* **11**, 3229. 10.1038/s41467-020-17038-2 (2020). 32591535 10.1038/s41467-020-17038-2 PMC7319989

[12] Werner, A. D. & Simmons, C. T. Impact of sea-level rise on sea water intrusion in coastal aquifers. *Groundwater* **47** (2), 197–204. 10.1111/j.1745-6584.2008.00535.x (2009). 10.1111/j.1745-6584.2008.00535.x 19191886

[13] Su, X., Befus, K. M. & Hummel, M. A. Shoreline barriers May amplify coastal groundwater hazards with sea-level rise. *Sci. Rep.* **14** (1), 15559. 10.1038/s41598-024-66273-w (2024). 38969675 10.1038/s41598-024-66273-w PMC11226656

[14] LeRoux, N. K., Frey, S. K., Lapen, D. R., Guimond, J. A. & Kurylyk, B. L. Mega-Tidal and Surface Flooding Controls on Coastal Groundwater and Saltwater Intrusion Within Agricultural Dikelands. *Water Resour. Res.* 59(11), e (2023). WR035054 10.1029/2023WR035054. (2023).

[15] Hu, S. et al. Impact of tidal dynamics and typhoon-induced inundation on saltwater intrusion in coastal farms. *Sci. Total Environ.* 91509. 10.1016/j.scitotenv.2024.170109 (2024). 10.1016/j.scitotenv.2024.170109 38232836

[16] Illangasekare, T. et al. Impacts of the 2004 tsunami on groundwater resources in Sri Lanka. *Water Resour. Res.* **42** (5), W05201. 10.1029/2006WR004876 (2006).

[17] Gaaloul, N., Pliakas, F., Kallioras, A., Schuth, C. & Marinos, P. Simulation of seawater intrusion in Coastal aquifers: Forty-five-years exploitation in an Eastern Coast aquifer in NE Tunisia. *Open. Hydrology J.* **6** (1), 31–44. 10.2174/1874378101206010031 (2012).

[18] Langevin, C. D. & Zygnerski, M. Effect of sea-level rise on salt water intrusion near a coastal well field in southeastern Florida. *Groundwater* **51** (5), 781–803. 10.1111/j.1745-6584.2012.01008.x (2013). 10.1111/j.1745-6584.2012.01008.x 23145832

[19] Sohrabi, M., Moftakhari, H. & Moradkhani, H. Efficient tropical cyclone scenario selection based on cumulative likelihood of potential impacts. *Earth’s Future*. **11** (10). 10.1029/2023EF003731 (2023). e2023EF003731.

[20] Therrien, R., McLaren, R. G., Sudicky, E. A. & Panday, S. M. *A three-dimensional Numerical Model Describing fully-integrated Subsurface and Surface Flow and Solute Transport* (Groundwater Simulation Group, 2010).

[21] Gholizadeh, H., Behrouj Peely, A., Karney, B. W. & Malekpour, A. Assessment of groundwater ingress to a partially pressurized water-conveyance tunnel using a conduit-flow process model: a case study in Iran. *Hydrogeol. J.* **28** (7), –2585. 10.1007/s10040-020-02213-y (2020).

[22] Ponprasit, C., Zhang, Y., Gu, X., Goodliffe, A. M. & Sun, H. Assessing vulnerability of regional-scale aquifer-aquitard systems in East Gulf coastal plain of Alabama by developing groundwater flow and transport models. *Water* **15** (10). 10.3390/w15101937 (2023).

[23] Yang, J., Graf, T. & Ptak, T. Sea level rise and storm surge effects in a coastal heterogeneous aquifer: a 2D modelling study in Northern Germany. *Grundwasser* **20**, 39–51. 10.1007/s00767-014-0279-z (2015).

[24] Holding, S. & Allen, D. M. Wave overwash impact on small islands: generalised observations of freshwater lens response and recovery for multiple hydrogeological settings. *J. Hydrol.* 529–1335. 10.1016/j.jhydrol.2015.08.052 (2015).

[25] Thornton, J. M., Therrien, R., Mariéthoz, G., Linde, N. & Brunner, P. Simulating fully-integrated hydrological dynamics in complex alpine headwaters: potential and challenges. *Water Resour. Res* **58**(4). https://doi.org/10.1029/2020WR029390 (2022). e2020WR029390.

[26] Stanic, S. et al. Saltwater intrusion into a confined Island aquifer driven by erosion, changing recharge, sea-level rise, and coastal flooding. *Water Resour. Res.* **60** (1). 10.1029/2023WR036394 (2024). e2023WR036394.

[27] Elsayed, S. M. & Oumeraci, H. Modelling and mitigation of storm-induced saltwater intrusion: improvement of the resilience of coastal aquifers against marine floods by subsurface drainage. *Environ. Model. Softw.* **100**, 252–277. 10.1016/j.envsoft.2017.11.030 (2018).

[28] Hochreiter, S. & Schmidhuber, J. Long short-term memory. *Neural Comput.* **9** (8), –1780. 10.1162/neco.1997.9.8.1735 (1997). 10.1162/neco.1997.9.8.1735 9377276

[29] Yu, Y., Si, X., Hu, C. & Zhang, J. A review of recurrent neural networks: LSTM cells and network architectures. *Neural Comput.* **31** (7), 1235–1270 (2019). 31113301 10.1162/neco_a_01199

[30] Shi, J., Wang, S., Qu, P. & Shao, J. Time series prediction model using LSTM-Transformer neural network for mine water inflow. *Sci. Rep.* **14**, 18284. 10.1038/s41598-024-69418-z (2024). 39112684 10.1038/s41598-024-69418-z PMC11306369

[31] Gholizadeh, H., Zhang, Y., Frame, J., Gu, X. & Green, C. T. Long short-term memory models to quantify long-term evolution of streamflow discharge and groundwater depth in Alabama. *Sci. Total Environ.*10.1016/j.scitotenv.2023.165884 (2023). 90184.3. 37517717 10.1016/j.scitotenv.2023.165884

[32] Oluwaniyi, O. et al. Correlating groundwater storage change and precipitation in alabama, united States from 2000–2021 by combining the water table fluctuation method and statistical analyses. *Sustainability* **15** (21), 4. 10.3390/su152115324 (2023).

[33] KarimiDermani, B. et al. Analyzing Multi-Year nitrate concentration evolution in Alabama aquatic systems using a machine learning model. *Environments* **12**(3), 75. 10.3390/environments12030075

[34] LeCun, Y., Bottou, L., Bengio, Y. & Haffner, P. Gradient-based learning applied to document recognition. *Proc. IEEE*. 86(11)-2324. (1998). 10.1109/5.726791

[35] Krizhevsky, A., Sutskever, I. & Hinton, G. E. Imagenet classification with deep convolutional neural networks. *Adv. Neural Inf. Process. Syst.* 25. 10.1145/3065386 (2012).

[36] Wang, Y., Fang, Z., Hong, H. & Peng, L. Flood susceptibility mapping using convolutional neural network frameworks. *J. Hydrol.* **582**, 124482. 10.1016/j.jhydrol.2019.124482 (2020).

[37] U.S. Geological Survey. USGS water data for the Nation: U.S. Geological Survey National Water Information System database, accessed [01/2025], at (2025). 10.5066/F7P55KJN

[38] Davis, M. E., Stratigraphic & Hydrogeologic Framework of the Alabama Coastal Plain; U.S Geological Survey Water-Resources Investigations Report 87-4112. and ; United States Department of the Interior, Geological Survey: Reston, VA, USA. (1987). 10.3133/wri874112

[39] Kratzert, F., Gauch, M., Nearing, G. & Klotz, D. NeuralHydrology—A Python library for Deep Learning research in hydrology. *J. Open Source Softw*. 7(71), p.4050. (2022).

[40] Nash, J. E. & Sutcliffe, J. V. River flow forecasting through conceptual models part I—A discussion of principles. *J. Hydrol.* **10** (3), 282–290. 10.1016/0022-1694(70)90255-6 (1970).

[41] Moriasi, D. N., Gitau, M. W., Pai, N. & Daggupati, P. Hydrologic and water quality models: performance measures and evaluation criteria. *Trans. ASABE*. **58** (6), –1785. 10.13031/trans.58.10715 (2015).

[42] Srivastava, A. et al. Compact and Generalizable Meta-LSTM Models for Memory Access Prediction In: Lauw, H., Wong, R.W., Ntoulas, A., Lim, E.P., Ng, S.K., Pan, S. (eds) Advances in Knowledge Discovery and Data Mining PAKDD 2020 Lect. Notes Comput. Sci., vol 12085 Springer, Cham. (2020). 10.1007/978-3-030-47436-2_5

[43] Kingma, D. P., Ba, J. & Adam: A method for stochastic optimization. arXiv preprint arXiv:1412.6980. https://doi.org/10.48550/arXiv.1412.6980 (2014).

[44] Torrence, C. & Compo, G. P. A practical guide to wavelet analysis. *Bull. Am. Meteorol. Soc.* **79** (1), 61–78. 10.1175/1520-0477(1998)079%3C0061:APGTWA%3E2.0.CO;2 (1998).

[45] Aquanty Inc. HydroGeoSphere user manual (p. 435). Waterloo. (2015).

[46] Reed, P. C. & McCain, J. F. *Water Availability of Baldwin County, Alabama Geological Survey of Alabama* (Division of Water Resources, 1971).

[47] Arnold, J. G., Srinivasan, R., Muttiah, R. S. & Williams, J. R. Large area hydrologic modeling and assessment part I: model development 1. *JAWRA J. Am. Water Resour. Assoc.* **34** (1), 73–89. 10.1111/j.1752-1688.1998.tb05961.x (1998).

[48] Saedi, F., Ahmadi, A. & Abbaspour, K. C. Optimal water allocation of the Zayandeh-Roud Reservoir in Iran based on inflow projection under climate change scenarios. *J. Water Clim. Change*, 12(5)- (2081). 10.2166/wcc.2021.219. (2021).

[49] Karamouz, M., Alipour, R. S., Roohinia, M. & Fereshtehpour, M. A. Remote sensing driven soil moisture estimator: uncertain downscaling with Geostatistically based use of ancillary data. *Water Resour. Res.* **58** (10). 10.1029/2022WR031946 (2022). e2022WR031946.

[50] Ponprasit, C. Quantifying groundwater storage and vulnerability for the state of Alabama using comprehensive flow and particle tracking models. Master’s Thesis, University of Alabama. (2023).

[51] Pilson, M. E. *An Introduction To the Chemistry of the Sea* (Cambridge University Press, 2012).

[52] Chandler, R. V., Moore, J. D. & Gillett, B. Ground-water chemistry and salt-water encroachment, Southern Baldwin county, alabama. alabama.Geol. *Surv. Bull.* **126**, 166 (1985).

[53] Ellis, J. Evaluation of submarine groundwater discharge and groundwater quality using a novel coupled approach: isotopic tracer techniques and numerical modeling. Master’s Thesis, The University of Alabama. (2013).

[54] Doherty, J. *PEST model-independent Parameter Estimation User Manual* (Watermark Numerical Computing, Brisbane, 2004).

[55] Gelhar, L. W., Welty, C. & Rehfeldt, K. R. A critical review of data on field-scale dispersion in aquifers. *Water Resour. Res.* **28** (7), 1955–1974. 10.1029/92WR00607 (1992).

[56] Harbaugh, A. W., Banta, E. R., Hill, M. C. & McDonald, M. G. Modflow-2000, the U.S. Geological Survey modular ground-water model—User guide to modularization concepts and the ground-water flow process. U.S. Geol. Surv. Open-File Rep. 00–92 (2000).

[57] Guo, W. & Langevin, C. D. *User’s Guide To SEAWAT: a Computer Program for Simulation of three-dimensional variable-density ground-water Flow (Vol. 1, No. 434)* (US Geol Surv, 2002).

[58] Glorot, X., Bordes, A. & Bengio, Y. Deep sparse rectifier neural networks. Proceedings of the fourteenth international conference on artificial intelligence and statistics (pp. 315–323). JMLR Workshop and Conference Proceedings. (2011).

[59] Reilly, T. E. & Harbaugh, A. W. Guidelines for evaluating ground-water flow models: *US. Geol. Surv. Sci. Inv. Rep.* 2004–5038, 30 p. (2004).

[60] U.S. Environmental Protection Agency. Water Quality Criteria, 1972. Washington, D.C., U.S. Government Printing Office, 594p. (1973).

[61] Huettemann, M. The effect of tropical storm systems on groundwater quality along coastal Alabama. Master’s Thesis, The University of Alabama. (2022).

[62] Chang, S. W., Clement, T. P., Simpson, M. J. & Lee, K. K. Does sea-level rise have an impact on saltwater intrusion? *Adv. Water Resour.* **34** (10), –1291. 10.1016/j.advwatres.2011.06.006 (2011).

[63] Custodio, E. Salt-fresh water interrelationships under natural conditions. Groundwater Problemsin Coastal Areas, UNESCO Studies and Reports in Hydrology, 45, 14–96. (1987).

[64] Coburn, N. L. Using electrical resistivity tomography to calibrate seawater intrusion models along the Alabama Gulf Coast. Master’s Thesis, The University of Alabama. (2015).

