# Supplementary material for: Modeling seawater intrusion along the Alabama coastline using physical and machine learning models to evaluate the effects of multiscale natural and anthropogenic stresses
Source: Sci Rep. 2025 Jul 1;15:21699. doi: 10.1038/s41598-025-06613-6 (PMC12218900; doi:10.1038/s41598-025-06613-6)
Supplement: Supplementary file 1 — Supplementary Material 1 [file 41598_2025_6613_MOESM1_ESM.docx]

**Supplementary Material** for “*Modeling seawater intrusion along the Alabama coastline using physical and machine learning models to evaluate the effects of multiscale natural and anthropogenic stresses*”

Hossein Gholizadeh^1^, T. Prabhakar Clement^2^, Christopher T. Green^3^, Geoffrey R. Tick^4^, Alain Plattner^1^, and Yong Zhang^1,*^

1. Department of Geological Sciences, University of Alabama, Tuscaloosa, AL 35487

2. Department of Civil, Construction, and Environmental Engineering, University of Alabama, Tuscaloosa, Alabama 35487

3. U.S. Geological Survey, Water Resources Mission Area, Moffett Field, CA 94035

4. Santa Clara Valley Water District, Groundwater Management Unit, San Jose, CA 95118

*Corresponding Author: Yong Zhang at yzhang264@ua.edu

## **Section A. Supplementary data**

**Table A1** provides details of the 15 observation wells (marked in **Fig. 1**), including their coordinates, water levels (annual average for 2023 in meter above sea level (masl)), depths, and the aquifers they penetrate. Hydraulic head data from these wells were used to construct the water table for the study area.

**Table. A1**. List of observation wells

| **No.** | **Well’s GSA ID** | **Water Level (masl)** | **Latitude** | **Longitude** | **Total Well Depth (m below well head)** | **Aquifer** | **Screen depth (m below well head)** | **Multi-level samplers** |
| --- | --- | --- | --- | --- | --- | --- | --- | --- |
| 1 | 92244 | 0.43 | 30.25003 | -87.6674 | 2.4 | CL1 | fully screened | No |
| 2 | 92272 | 1.47 | 30.28045 | -87.6498 | 110 | CL2 | 40 - 110 | Yes |
| 3 | 92278 | 1.83 | 30.28045 | -87.6498 | 40.0 | CL1 | fully screened | No Info. |
| 4 | 92287 | 0.43 | 30.23097 | -87.8319 | 1.6 | CL1 | fully screened | No |
| 5 | 92322 | 7.17 | 30.34707 | -87.6682 | 68.0 | CL2 | 40 -68 | No Info. |
| 6 | 92347 | 2.13 | 30.29928 | -87.6474 | 15.2 | CL1 | fully screened | No |
| 7 | 92379 | 1.68 | 30.28797 | -87.6333 | 151.2 | CL3 | 90 – 151.2 | Yes |
| 8 | 92389 | 9.84 | 30.37113 | -87.6849 | 18.3 | CL1 | fully screened | No |
| 9 | 92395 | 1.52 | 30.28812 | -87.6332 | 36.6 | CL1 | fully screened | No Info. |
| 10 | 92400 | 15.87 | 30.47227 | -87.8839 | 102.4 | CL3 | 80 – 102.4 | No Info. |
| 11 | 92197 | 15.71 | 30.40857 | -87.6785 | 36.6 | CL1 | fully screened | No Info. |
| 12 | 92419 | 1.44 | 30.28696 | -87.6500 | 21.3 | CL1 | fully screened | No |
| 13 | 143713 | 0.4 | 30.2294 | -88.0243 | 1.3 | CL1 | fully screened | No |
| 14 | 143715 | 0.49 | 30.25122 | -87.6672 | 3.4 | CL1 | fully screened | No |
| 15 | 92841 | 2.5 | 30.27872 | -87.6662 | 39.6 | CL1 | fully screened | Yes |

**Table A2** shows salinity measurements for groundwater collected at nine wells.

**Table A2.** Location coordinates and depth of wells with salinity data used in the HGS model.

| **No.** |  | **Latitude** | **Longitude** | **Well Depth (m below well head)** | **Screen depth (m below well head)** |
| --- | --- | --- | --- | --- | --- |
| 1 |  | 30.24983 | -87.6674 | 2.5 | fully screened |
| 2 |  | 30.25103 | -87.6673 | 3.5 | fully screened |
| 3 |  | 30.25369 | -87.6906 | 7.5 | fully screened |
| 4 |  | 30.2545 | -87.6935 | 23.0 | fully screened |
| 5 |  | 30.2589 | -87.6947 | 9.0 | fully screened |
| 6 |  | 30.26238 | -87.6874 | 32.0 | fully screened |
| 7 |  | 30.27472 | -87.6861 | 20.0 | fully screened |
| 8 |  | 30.2776 | -87.6886 | 1.5 | fully screened |
| 9 |  | 30.28208 | -87.6859 | 20.0 | fully screened |

**Fig. A1** scatterplot comparing simulated versus observed groundwater depth, used to evaluate the LSTM model performance.


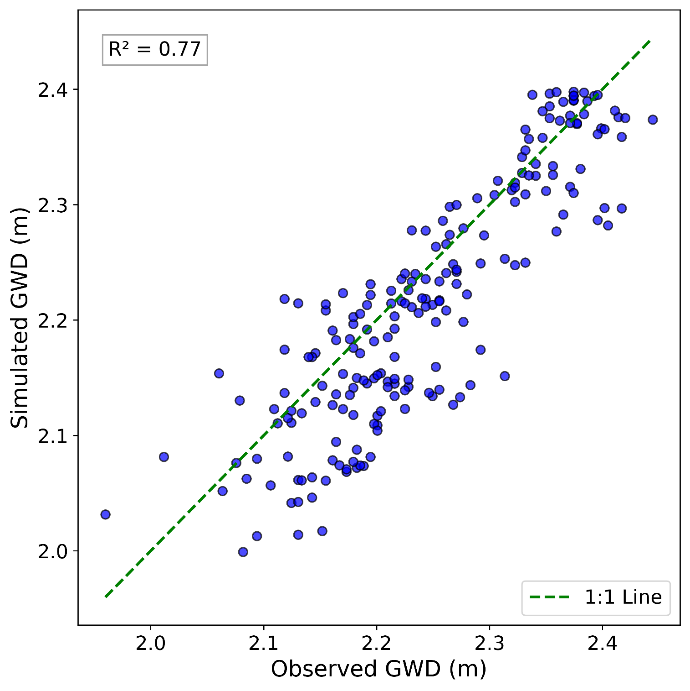


**Fig. A1 (a).** Simulated versus observed groundwater depth for well 92841 (CL1 aquifer).


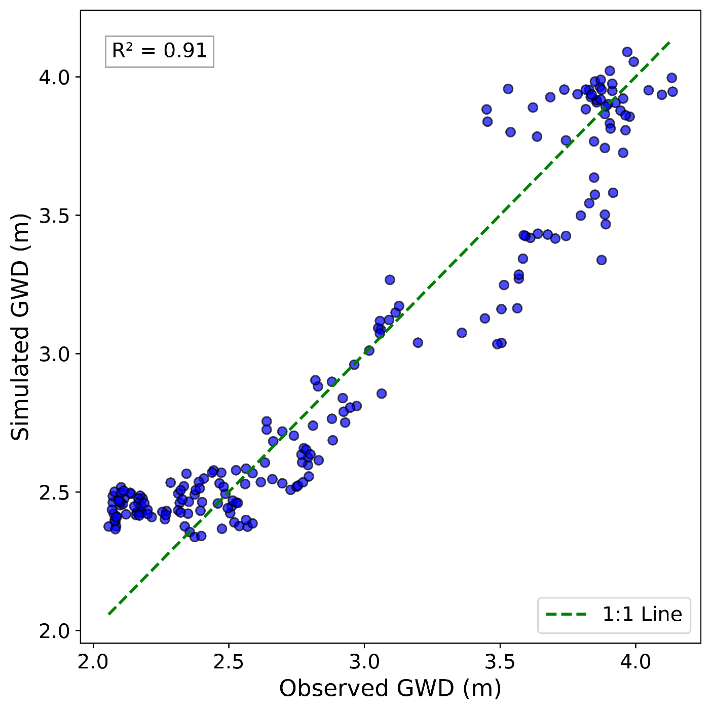


**Fig. A1 (b).** Simulated versus observed groundwater depth for well 92272 (CL2 aquifer).

**Fig. A2** presents long- and short-term fluctuations in sea levels based on NOAA’s [National Ocean Service](http://oceanservice.noaa.gov/) data.


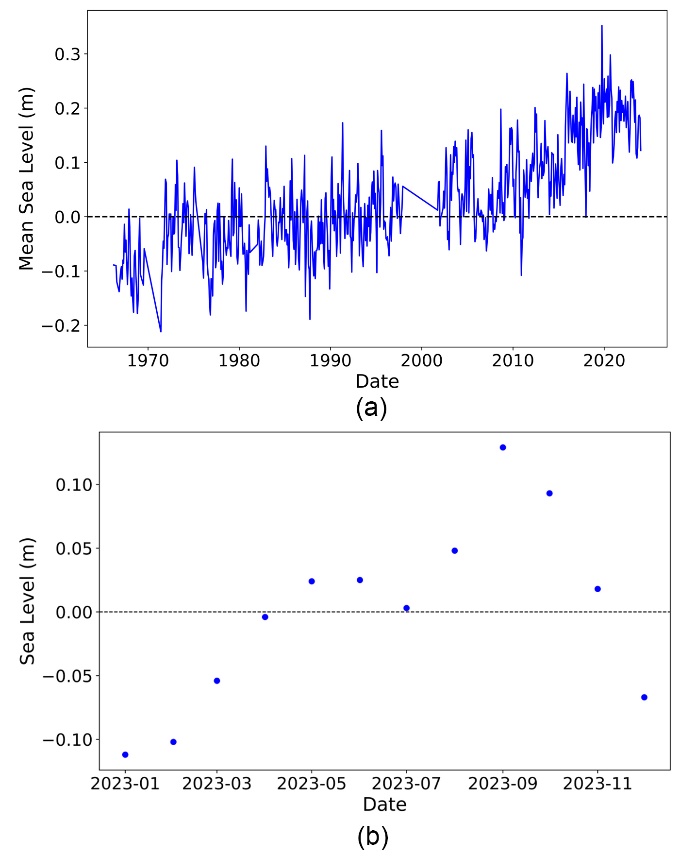


**Fig. A2.** Long-term sea level variations (1967-2023, with gaps) (a) and monthly variations in 2023 (b) at Dauphin Island, Alabama (NOAA/National Ocean Service, https://tidesandcurrents.noaa.gov/sltrends/sltrends_station.shtml?id=8735180).

## **Section B. SEAWAT Model Description**

To generate a seawater intrusion probability map for the study area, we developed a SEAWAT model using the GMS interface for MODFLOW. The model domain, shown in Fig. 1, spans 48,113 meters along the *x*-axis and 30,125 meters along the *y*-axis, with a vertical thickness of approximately 150 meters. The aquifer system was discretized into three hydrostratigraphic layers — CL1, CL2, and CL3 (from top to bottom)—based on regional geological data.

The horizontal model grid consists of 200 columns (*x*-direction) and 300 rows (*y*-direction), totaling 60,000 horizontal cells with uniform horizontal cell dimensions of approximately 240.6 × 100.4 meters.

Hydraulic conductivity values were assigned as 1 × 10⁻³ m/s for CL1, 6 × 10⁻⁵ m/s for CL2, and 1 × 10⁻⁵ m/s for CL3. A recharge rate of 9 × 10⁻⁴ m/day was applied across the land surface. The specific yield for the unconfined upper layer (CL1) was set to 0.3, while storage coefficients of 0.00014 and 0.00006 were assigned to the confined layers CL2 and CL3, respectively.

Flow boundary conditions consisted of constant head boundaries along the southern and western edges of the domain, and variable head boundaries along the northern and eastern edges to represent inland hydraulic gradients.

Solute transport was simulated using the MT3DMS module. The model was initialized with freshwater conditions (0 mg/L) throughout the domain, except at the seawater boundaries. Constant concentration boundary conditions of 35,000 mg/L were applied along the southern and western edges to represent seawater. Variable-density flow was activated using the VDF package, with fluid densities set to 1,025 kg/m³ for seawater and 1,000 kg/m³ for freshwater.

The model was run over a simulation period of 10 years using daily stress periods to capture temporal variations in flow and transport. This model configuration was designed to generate input data for training the CNN model.
